# First Characterization of *Acinetobacter baumannii*-Specific Filamentous Phages

**DOI:** 10.3390/v16060857

**Published:** 2024-05-27

**Authors:** Jelena Narancic, Damir Gavric, Rok Kostanjsek, Petar Knezevic

**Affiliations:** 1 PK Lab., Department of Biology and Ecology, Faculty of Sciences, University of Novi Sad, 21000 Novi Sad, Serbia; jelenan@dbe.uns.ac.rs (J.N.); damir.gavric@dbe.uns.ac.rs (D.G.); 2 Department of Biology, Biotechnical Faculty, University of Ljubljana, Jaminkarjeva 101, SI-1000 Ljubljana, Slovenia; rok.kostanjsek@bf.uni-lj.si

**Keywords:** filamentous phage, *Acinetobacter baumannii*, growth kinetics, twitching motility, biofilm, antibiotic susceptibility, efflux pump

## Abstract

Filamentous bacteriophages belonging to the order *Tubulavirales*, family *Inoviridae*, significantly affect the properties of Gram-negative bacteria, but filamentous phages of many important pathogens have not been described so far. The aim of this study was to examine *A. baumannii* filamentous phages for the first time and to determine their effect on bacterial virulence. The filamentous phages were detected in 15.3% of *A. baumannii* strains as individual prophages in the genome or as tandem repeats, and a slightly higher percentage was detected in the culture collection (23.8%). The phylogenetic analyses revealed 12 new genera within the *Inoviridae* family. Bacteriophages that were selected and isolated showed structural and genomic characteristics of the family and were unable to form plaques. Upon host infection, these phages did not significantly affect bacterial twitching motility and capsule production but significantly affected growth kinetics, reduced biofilm formation, and increased antibiotic sensitivity. One of the possible mechanisms of reduced resistance to antibiotics is the observed decreased expression of efflux pumps after infection with filamentous phages.

## 1. Introduction

All filamentous bacteriophages discovered so far belong to the order *Tubulavirales* (realm *Monodnaviria*, kingdom *Loebvirae*, phylum *Hofneiviricota*, class *Faserviricetes*). The *Inoviridae* family is classified under the *Tubulavirales* order, alongside the *Plectroviridae* and *Paulinoviridae* families [1]. The family comprises over 20 genera, with many of them consisting of a single species. Their virions are long rigid or flexible filaments containing circular, single-stranded DNA (ssDNA). Filamentous phages are not typical obligatory lytic or temperate phages, as they undergo neither the typical lytic nor lysogenic cycle. Inoviruses attach to bacterial pili and deliver their DNA into the cell. Filamentous phages can exist in the form of prophages, integrated into the bacterial genome, or persist episomally. For replication, they use a rolling-circle mechanism or transposition, and virions assemble on the cell membrane, extruding through the bacterial cell wall. In this manner, cell damage and bacterial lysis are avoided, and filamentous phages establish chronic productive infections, continuously releasing new virions. In Escherichia phage M13, virions are made of a major coat protein in 2700 copies (CoaB, p8) helically organized around a circular, positive, ssDNA and minor coat proteins with 5 copies each (CoaA or p3; p6; p7; p9) [2]. The replication genes (*g2*, *g5*, and *g10*), structural genes (*g7*, *g9*, *g8*, *g3*, and *g6*), and morphogenesis genes (*g1*, *g4* and *g11*) are organized into three modules in the *Inoviridae* genome. Sufficiently conserved proteins among the family *Inoviridae* are major coat protein (CoaB) and morphogenesis protein (Zot; zonula occludens toxin). Zot protein has an ATPase function in the conserved part, and in some cases, it is implicated in the virulence of pathogenic host bacteria.

Inoviruses represent a significant group of bacteriophages due to their potential impact on bacterial virulence. These viruses have been shown to impact the growth rate of their bacterial hosts [3,4,5,6]. It has been postulated that the exceptionally high production of these phages may lead to an overabundance of copies of the major coat protein on the surface of bacterial cells, potentially causing this phenomenon. Additionally, the accumulation of filamentous phages on the surface of bacterial cells can significantly affect aggregation [7] and the hydrophobicity of cells [8]. In certain instances, changes in bacterial motility have been observed following infection with filamentous phages. Filamentous phages have been demonstrated to inhibit both swarming and swimming motilities in various studies [9,10,11]. Bacteria utilize pili for twitching motility, which is also frequently inhibited by filamentous phages [10,11,12]. These phenotypic changes ultimately impact the initial stages of biofilm development. In a previous study, we successfully demonstrated that Pf4 phages influence numerous virulence factors of newly infected *P. aeruginosa* strains, primarily by reducing virulence, with the exception of swimming motility and biofilm production [13]. One of the most intriguing filamentous phages is undoubtedly CTXϕ of *Vibrio cholerae* bacteria, which carries genes encoding three toxins associated with cholera: CT (cholera toxin), Zot (zonula occludens toxin), and Ace (accessory cholera toxin) [14,15,16]. Currently, only a small number of inoviruses have been described, i.e., phages infecting the following Gram-negative genera have been examined: *Escherichia*, *Salmonella*, *Pseudomonas*, *Xanthomonas*, *Stenotrophomonas*, *Vibrio* and *Ralstonia*. There are currently 43 species and 25 genera approved [17], but analyses of bacterial genomes indicate a much higher prevalence of these prophages in bacteria [1,18,19]. It is important to note that filamentous bacteriophages of some important pathogens, such as the emerging opportunistic pathogen *Acinetobacter baumannii*, have not been investigated so far, despite their significant role in bacterial pathogenicity.

*A. baumannii* is a strictly aerobic Gram-negative coccobacillus that is non-motile, catalase-positive, and oxidase-negative. It is commonly found in various environments such as soil, water, and food products, and it can also be part of the human microbiota. It can survive on surfaces for several days and withstand drying and disinfection processes [20]. *A. baumannii* is an etiological agent of various infections, and it is frequently isolated from medical devices, wounds, blood, sputum, bronchial rinsing, urine, and catheter drains [20,21,22,23]. Moreover, *A. baumannii* is a nosocomial pathogen with high mortality in intensive care settings, as it belongs to the group of most resistant bacteria today (ESCAPE group), frequently showing multidrug or even pan-drug resistance [24,25,26,27]. According to evidence, meningitis, wounds, pneumonia (hospital and community-acquired), bacteraemia, burns, endocarditis, urinary tract infections (UTIs) and skin and soft tissue infections are the most prevalent *Acinetobacter*-associated nosocomial illnesses [28,29].

Taking into account the medical importance of *A. baumannii* and the lack of data regarding their filamentous phages, the aim of this study was to determine the prevalence of filamentous phages among *A. baumannii*; isolate and characterize *A. baumannii*-specific filamentous phages; and to examine their role in bacterial virulence.

## 2. Materials and Methods

### 2.1. Bacterial Strains

In the study, 31 previously described strains [30,31], including reference ones—ATCC 19606, ATCC BAA747, and NCTC 13423 of *A. baumannii* deposited at PK Lab (Department of Biology and Ecology, Faculty of Science, University of Novi Sad, Novi Sad, Serbia)—were used (Table S1). Also, 32 strains newly isolated from surface water for the purposes of this study were used. The strains were initially identified through biochemical tests and subsequently confirmed using PCR with *A. baumannii*-specific primer pairs: P-Ab-ITSF and P-Ab-ITSR specific for the genus, and P-rA-1 and P-rA-2 for the species (Table S2). All *A. baumannii* strains, 63 in total, were preserved as glycerol stocks at a temperature of −80 °C and cultured on Mueller-Hinton agar overnight at 37 °C. Further cultivation procedures involved the utilization of various media, such as Mueller–Hinton (MH) broth or Lysogeny Broth (LB) agar with different agar concentrations and brain heart infusion broth (BHI), depending on the specific experimental requirements.

### 2.2. In Silico Identification and Classification of Filamentous Prophages in A. baumannii Strains

The available genome of a reference strain ATCC 19606 (Genome Access. No.NZ_CP059040.1/CP059040.1) was examined for the presence of inovirus prophages by BLAST algorithm and using PHASTER platform (PHAge Search Tool Enhanced Release) [32]. PHASTER serves as a web-based tool designed for swift identification and annotation of prophage sequences present in bacterial genomes. Using this platform, a sequence similar to filamentous phages was found in the ATCC 19606 genome, and the prophage sequence was analyzed to determine genome structure. The phage was designated as Acinetobacter phage Af1, while all other *A. baumannii*-specific filamentous phages we found were designated in this study as Af.

The sequences of *zot*, *coaA*, and *coaB* genes from the Af1 filamentous prophage were utilized to search by BLASTn for other Af prophages in a total of 541 *A. baumannii* genome sequences deposited in GenBank (Table S3) [33]. Based on amino-acid sequences of Zot of all Af prophages found, a phylogenetic tree was constructed, and prophages were divided into groups designated by letters, from A to J. Amino-acid sequence alignment was performed in MEGA X [34]. The phylogenetic tree was constructed using the maximum likelihood method and the Jones–Taylor–Thornton (JTT) model. The identified Zot and CoaB were analyzed in accordance with ICTV recommendations, to determine possible genera [1]. 

### 2.3. PCR Identification of Filamentous Phages in Different A. baumannii Strains

The bacterial DNA was isolated from overnight cultures of *A. baumannii* strains using a commercial GeneJET Genomic DNA Purification Kit (Thermo Fisher Scientific, Vilnius, Lithuania). Degenerate primers were designed for the *zot* gene, targeting phage groups previously defined based on the specific amino-acid sequences of Zot (Table S2). The quality of the designed primers was assessed using OligoAnalyzer 3.1 [35] and PCR Primer Stats [36]. Two more pairs of primers specific for *A. baumannii* genus and species were used as a positive control, and distilled water was used as a negative. The isolated genomic DNA of each *A. baumannii* strain was used as a template for PCR. The products were analyzed by agarose gel electrophoresis with ethidium bromide and visualized under UV light with a 100 bp marker (BioDocAnalyze System, Biometra, Gottingen, Germany). 

### 2.4. Propagation and Purification of A. baumannii-Specific Filamentous Phages

One reference strain, ATCC 19606, the clinical strain Aba-4804 and the environmental strain Aba-K, containing the most prevalent genetic elements of Af filamentous prophages, were used for the isolation and propagation of *A. baumannii*-specific filamentous virions. To isolate integrated filamentous phages, a method recently described by Gavric and Knezevic (2022a) was used [37]. Briefly, 600 mL of LB broth was inoculated with 1 mL of overnight bacterial cultures. Incubation was carried out at 37 °C with shaking at 250 rpm for 48 h. After two days of incubation, bacteria were removed from the liquid cultures through multiple centrifugation steps. Potentially released filamentous bacteriophages were retained in the supernatant. Then, fresh medium was added to the initial volume, and a new overnight culture was inoculated. These steps were repeated after the fourth day of incubation. After the sixth day of incubation, liquid cultures were centrifuged, and the phages from the supernatant were precipitated overnight at 4 °C with 4% polyethylene glycol 8000 (PEG8000) and 0.5 M NaCl. The following day, tubes were centrifuged, and the pellet was resuspended to a total volume of 6 mL of SM buffer [38], concentrating a starting volume of 600 mL 100 times. 

The virions were further purified in CsCl equilibrium (0.375 g mL^−1^ caesium chloride, Sigma-Aldrich, Saint Louis, MO, USA) for 42 h at 4 °C by centrifugation at 135,000× *g* (fixed angle rotor, Ti50, Beckman Coulter, Brea, CA, USA). The obtained suspension was dialyzed in 2L SM buffer for each milliliter of phage suspension and used to further characterize *A. baumannii* phages.

### 2.5. Transmission Electron Microscopy (TEM) Imaging of Isolated A. baumannii-Specific Filamentous Phages

The suspensions of phages were negatively stained using a droplet method for transmission electron microscopy [39]. In order to allow viral particles to adhere to the Formvar film, suspensions were placed onto copper grids that had been previously coated with carbon and left to dry for five minutes. The attached viral particles were immediately stained with an aqueous solution of uranyl acetate (0.5–1% *w*/*v*) for one minute after the excess suspension was removed. Then, after being air-dried, stained samples were examined using an 80 kV Philips CM 100 transmission electron microscope (Philips, Amsterdam, The Netherlands). The images were captured using the Digital Micrograph 3.4 program and a Gatan Bioscan CCD camera (Gatan Inc., Pleasanton, CA, USA). A minimum of 10 measurements of the phage’s morphological characteristics were averaged.

### 2.6. Protein Characterization of A. baumannii-Specific Filamentous Phages

The SDS-PAGE method was used for the determination of protein profiles of CsCl-purified virions [40]. Protein extraction from phage suspensions was performed using the chloroform-methanol method [41]. Phage suspension, ice-cold methanol, and chloroform were mixed in a ratio of 1:1:0.75 and further centrifuged 18,000× *g* for 10 min at 4 °C. The upper phase of the chloroform-containing mixture was separated. An equal volume of fresh methanol was added, followed by another centrifugation step. The upper layer of methanol was discarded, and the methanol treatment step was repeated to obtain precipitated phage proteins.

The precipitate was dissolved in 50 μL of distilled water, and the concentration of proteins was quantified using the Bradford protein assay [42]. Proteins at the desired concentration were combined with 2× SDS-PAGE sample buffer (*v*/*v* 1:1), heated at 98 °C for 5 min, and promptly cooled on ice. The resulting protein samples were subsequently subjected to analysis using SDS-PAGE. Samples were run on a polyacrylamide gel, at 200 V in a running buffer (Tris-HCl 0.25 mol L^−1^; glycine 0.19 mol L^−1^; SDS 0.1% *w*/*v*, pH 8.65). The resolving part of the gel was 15% *v*/*v*, and the stacking part had a lower acrylamide concentration (~4% *v*/*v*). The PageRuler™ Unstained Low Range Protein Ladder (Thermo Fisher Scientific, Vilnius, Lithuania) was used for the determination of the molecular mass of the virion proteins. After electrophoresis, the gel was fixed in 5% *w*/*v* glutaraldehyde in order to preserve proteins of small molecular mass. After that, the gel was fixed in methanol-glacial acetic acid solution (50% MeOH and 10% glacial acetic acid), after which it was stained in Commasie Brilliant Blue solution, discolored, and documented by BioDocAnalyze 2.2 (Biometra, Gottingen, Germany).

To predict the in silico size of CoaA andCoaB, SignalIP—5 was used [43] to remove the signal sequence of the protein, which is not part of the virions.

### 2.7. Viral DNA Isolation and Treatment with Different Enzymes

Viral DNA isolation was performed using the phenol-chloroform method. To remove residual bacterial DNA and RNA in the phage suspension, 5 U mL^−1^ of DNase, 10 μg mL^−1^ of RNase, and 1.5 mM mL^−1^ of MgCl_2_ buffer were added. Samples were left for 24 h at 37 °C. After incubation, DNase and RNase were inactivated by incubation at 65 °C for 1 h. Then, 20 mM of Na_2_EDTA, 50 µL mL^−1^ of proteinase K, and 0.5% SDS were added, and the mixture was incubated at 56 °C for 1 h. The phage DNA was extracted using the phenol-chloroform method, followed by ethanol precipitation. The DNA was initially precipitated with twice the volume of ice-cold 96% ethanol and 0.1 volume of 3M sodium acetate at −20 °C for 30 min. The precipitated DNA was then purified using ice-cold 70% ethanol. The resulting pellet was dried and dissolved in Elution Buffer (Mini Prep 10 mM Tris-HCl, pH 8.5).

To confirm that the isolated viral DNA belonged to filamentous phages and was circular single-stranded DNA, the following enzymes were used for verification: DNase, TaqI, S1 nuclease, and ExoI nuclease. The number of potential dsDNA fragments produced by TaqI was predicted using the WebCutter tool [44]. The isolated viral DNA was also examined for the presence of the *zot* gene, a highly conserved gene of filamentous phages, and for the presence of genes specific to the genus and species of the *A. baumannii* bacterium, as a control.

### 2.8. Assessment of Filamentous Phage Plaque Formation by SPOT Methods

Due to the distinctive characteristics of filamentous phages and their inability to form plaques, both single- and double-layer SPOT methods were used to evaluate the isolated phages’ capacity to inhibit bacterial growth and subsequently form plaques [35]. Phage suspensions were prepared in SM buffer as ten-fold dilutions from 10^−1^ to 10^−10^, and 10 µL from each dilution was added to bacterial lawns of 63 *A. baumannii* strains and put on incubation at 37 °C for 24 h. To potentially enhance plaque formation, the SPOT method was combined with 1/4 MIC and 1/8 MIC of ceftriaxone, ciprofloxacin, chloramphenicol, gentamicin, kanamycin, polymyxin B, and tetracycline for each strain.

### 2.9. A. baumannii-Specific Filamentous Phage Infection of Selected Strains

Filamentous phages Af1, Af4, and AfK isolated from ATCC 19606 (group A), Aba-4804 (group A), and Aba-K strains (group C) were utilized for the infection of a total of 43 selected *A. baumannii* strains, which were negative for PCR targeting the *zot* gene, indicating the absence of *A. baumannii*-specific filamentous prophages. The infection of *A. baumannii* strains with filamentous phages was performed using the method described by Gavric and Knezevic (2022b) [13]. The presence of Af1, Af4, and AfK phages in *A. baumannii* strains was confirmed by the PCR method with specific primers (Table S2) and the sequencing of the obtained product. Thermal cycling conditions were as follows: an initial cycle of 94 °C for 5 min followed by 35 cycles of 94 °C for 30 s, annealing at 58 °C for 20 s, and extension at 72 °C for 60 s, with a final 7 min extension at 72 °C. PCR results were observed by gel electrophoresis on a 2% agarose gel stained with ethidium bromide (BioDocAnalyze System, Biometra, Gottingen, Germany).

### 2.10. A. baumannii-Specific Filamentous Phages Influence on Host Phenotype

#### 2.10.1. Growth Kinetics

Bacterial suspensions of 3 McFarland density (~9 × 10^8^ CFU mL^−1^) were made using overnight bacterial cultures and further diluted in a double-concentrated MH broth at a ratio of 1:1 (*v*/*v*) before being diluted in PBS [38] at a ratio of 1:100 (*v*/*v*). Additionally, to the wells of a microtiter plate, 200 µL of inoculated medium was added in duplicates. During the incubation for 24 h at 37 °C with constant shaking in a spectrophotometer (Multiscan GO, Thermo Fisher Scientific, Vantaa, Finland) every 30 min, the absorbance was measured at OD_600_, and the average values for the infected strains were compared to those of the uninfected strains. Results were averaged across the three independent replicates of the experiment.

#### 2.10.2. Twitching Motility

A twitching motility test was performed by stabbing *A. baumannii* infected and non-infected strains in LB medium plates with 1.5% agar. Following a 24 h incubation at 37 °C, the agar was removed, and a 0.4% crystal violet solution was applied to each Petri dish. This test was performed in four independent repetitions. Tested *A. baumanni* strains were classified using existing criteria for twitching motility for this species: <5 mm non-motile, 5–20 mm intermediate, and >20 mm highly motile [45].

#### 2.10.3. Biofilm Formation on Polystyrene Surface

In order to observe the influence of Af phages on *A. baumannii* biofilm formation, the method described for *P. aeruginosa* was used [46]. The overnight bacterial cultures of infected and non-infected strains were centrifuged at 6000× *g* for 2 min. The resulting supernatant was removed, and the pellet was resuspended in 1 mL of PBS buffer and centrifuged again. These steps were repeated twice and then the bacterial suspensions of 0.5 McFarland density (~10^8^ CFU mL^−1^) were prepared. Suspensions were diluted in PBS at a ratio of 1:100 (*v*/*v*) and then further diluted in MH broth in a ratio of 1:1 (*v*/*v*). A volume of 300 µL of the inoculated medium was dispensed into the wells of a microtiter plate and then incubated at 37 °C for 24 h. After incubation, the medium was carefully removed, and wells were washed twice with PBS. The absolute methanol was added for the fixation of the formed biofilm. After 15 min, methanol was removed, and the plate was allowed to dry for 15 min at 44 °C. Further, the biofilm was stained by 0.4% crystal violet for 15 min, and then the microtiter plate was submerged in tap water to remove excess crystal violet. After the plate had dried, 300 µL of 33% acetic acid was added to dissolve the fixed stain, and the microtiter plate was then kept at room temperature for 20 min. A spectrophotometer (Multiscan GO, Thermo Fisher Scientific, Vantaa, Finland) was used to measure the optical density for each well at 595 nm. Using the ODc value, the assessment of cell adhesion was conducted. The ODc value demonstrates the mean optical density of the negative control (OD) for the tested microtiter plate; it increased by three standard deviations obtained for the negative controls. The following standards were used for group *A. baumannii* strains: OD ≤ ODc = unadherent; ODc < OD ≤ (2 × ODc) = poorly adherent; (2 × ODc) < OD ≤ (4 × ODc) = moderately adherent (4 × ODc) < OD = highly adherent [47]. The values obtained for infected strains were averaged and compared to their uninfected strain equivalents. This test was run three times independently in triplicates.

#### 2.10.4. Carbohydrate Production

The total amount of polysaccharides in bacterial cells, including lipooligosaccharides (LOS) and capsular polysaccharides, were quantified in order to determine the Af effect on capsule production. Six bacterial strains, uninfected and infected with Af4 and AfK phages, were cultivated in 50 mL BHI broth for five days at 37 °C to stimulate carbohydrate production. Strain ATCC 19606 was used as a control since it belongs to non-capsulated strains. Cultures were then centrifuged for 5 min at 14,000× *g*. The extraction process involved washing the cells five times with 10 mL of 50 mM NaCl, with centrifugations for 5 min at 14,500× *g*. The cells were then resuspended in 50 mM EDTA and normalized in order to bring the OD600 equal to 0.25. Normalized suspensions were incubated for 1 h at 37 °C to allow the polysaccharides to be released from the cell. One more centrifugation was performed at the same conditions, and supernatant was collected in a new cuvette. After extraction of polysaccharides, 200 µL of each strain was utilized for measurement. The carbohydrate concentrations were determined using the sulfuric acid/phenol basis method, with yellow-colored furfural formation [48,49]. A standard curve was created using a range of known sucrose/fructose (1:1 *W*/*W*) concentrations (0–120 μg/mL). To all 200 µL volume samples, standards and EDTA as a negative control, 200 μL of 5% phenol (in water) and 1 mL of 93% sulfuric acid were added and left at room temperature for 10 min with occasional stirring. The resulting furfural derivatives were quantified using a spectrophotometer (Multiscan GO, Thermo Fisher Scientific, Vantaa, Finland) by measuring optical density at 490 nm for each well. Using the standard curve and interpolating values with linear regression, the total amount of carbohydrates was calculated.

#### 2.10.5. Antibiotic Susceptibility

Bacterial suspensions of 0.5 McFarland value (~10^8^ CFU mL^−1^) were prepared and diluted in MH broth (1:100 *v*/*v*). The final concentration of ceftriaxone (CTX), tobramycin (TOB) or ciprofloxacin (CIP) in the microtiter plates ranged from 0.125 to 512 µg mL^−1^ and was applied to the wells of a microtiter plate along with the same volume of inoculated medium. A total of ~1 × 10^6^ CFU mL^−1^ of bacteria were detected in each well of the microtiter plate. The microtiter plates were then incubated for 18 h at 37 °C, and the 2,3,5-triphenyltetrazolium chloride (TTC) solution was added to each well of the microtiter plates in 10 µL of a 0.1% solution, where it was converted to red formazan by the dehydrogenases of living bacterial cells. The MIC for each antibiotic was then read after the microtiter plates had been incubated for an additional two hours at 37 °C. The MIC value was defined as the lowest concentration of antibiotic required to prevent TTC from becoming red formazan. As a control, *Escherichia coli* ATCC 25922 was used, and at least three separate triplicates of the experiment were completed. According to the approved criteria for *A. baumannii*, infected and uninfected strains have been classified as sensitive, intermediate resistant, or resistant to antibiotics [50]. The results are shown as a geometric mean. A significant change was defined as a two-value reduction in the MIC for infected strains or a change in the qualitative sensitivity (sensitive, intermediate resistant, or resistant).

#### 2.10.6. Expression of Efflux Pumps

The qRT-PCR method was used to check the changes in expression of the following efflux pumps: *adeA*, *adeB*, *adeC*, *abeMRT* after filamentous phage infection. LB broth in a volume of 50 mL was inoculated with overnight culture of infected and non-infected strains (~10^6^ CFU mL^−1^) and incubated at 37 °C with orbital shaking at 200 rpm for 6 h. After incubation, 1 mL of inoculated medium was used to isolate RNA using the GeneJET RNA Purification Kit (Thermo Fisher Scientific, Vilnius, Lithuania). The isolated RNA from all samples was equilibrated and then purified with 1 U of DNAse I for 30 min at 37 °C. The purified RNA samples were translated into cDNA using a High-Capacity cDNA Reverse Transcription Kit (Applied Biosystems, Bedford, MA, USA) with the following steps: 10 min at 25 °C, then 120 min at 37 °C, and finally 5 min at 85 °C. The reaction mixture was cooled to 4 °C, and SYBR Green-based qRT-PCR with six primer pairs specific to the mentioned efflux pumps (Table S2) was performed. To normalize data, a primer pair targeting the *A. baumannii rpoB* housekeeping gene was used. Relative gene expression (RFC) was determined by 2^−(ΔΔCt)^, with a cut-off RFC value < 0.67 for decreased expression and >1.5 for overexpression.

## 3. Results

### 3.1. Identification and Classification of Filamentous Prophages in A. baumannii Strains

**Figure 1:**
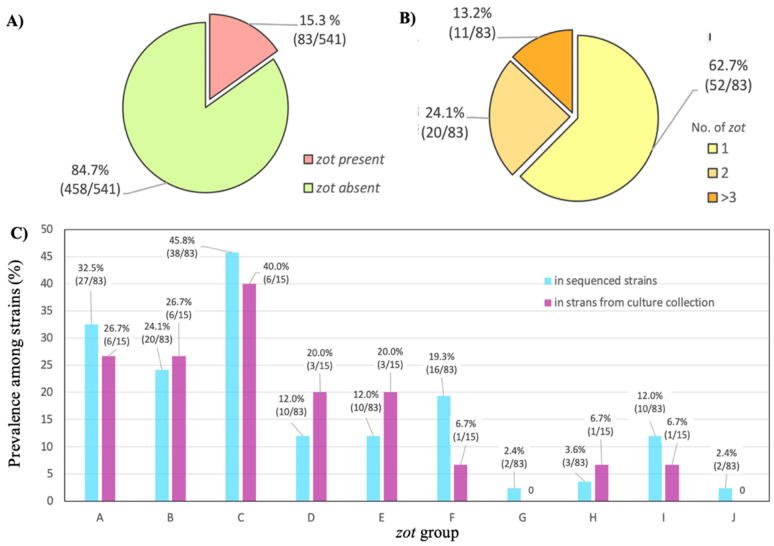
Prevalence of Af phages in *A. baumannii*: (**A**) Prevalence of prophages among *A. baumannii* determined in silico using GenBank deposited genomes, expressed as a percentage; (**B**) Prevalence of prophages among Af-positive strains, with various numbers of *zot* genes; (**C**) Prevalence of *zot* gene groups obtained in silico and by PCR in culture collection.

**Figure 2:**
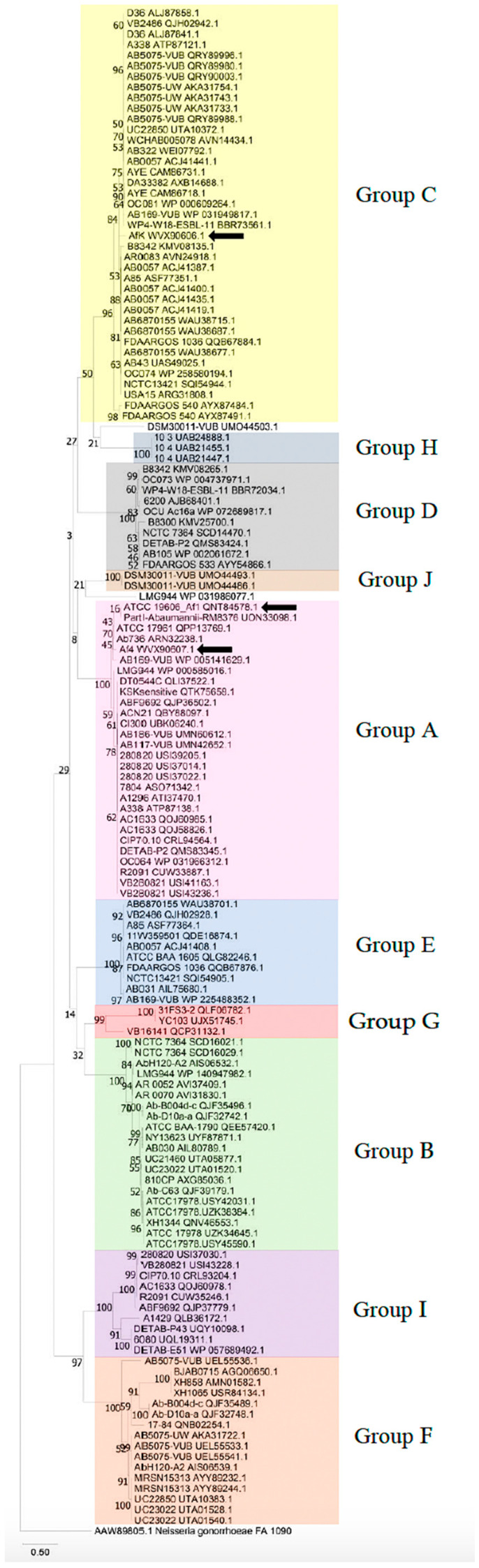
Phylogenetic tree of *A. baumannii*-specific filamentous phages, groups A–J based on amino-acid sequences of Zot proteins; two strains (DSM30011-VUB and LMG944) do not belong to a specific group. Zot of AfK, Af1 and Af4 are indicated by the black arrows. The evolutionary history was inferred by using the maximum likelihood method and JTT matrix-based model [51]. The tree with the highest log likelihood (−20,514.03) is shown. The percentage of trees in which the associated taxa clustered together is shown next to the branches. Initial tree(s) for the heuristic search were obtained automatically by applying Neighbor-Join and BioNJ algorithms to a matrix of pairwise distances estimated using the JTT model and then selecting the topology with superior log likelihood value. The tree is drawn to scale, with branch lengths measured in the number of substitutions per site. This analysis involved 142 amino-acid sequences. Zot of *Neisseria gonorrhoeae* was used as an outlier. There was a total of 672 positions in the final dataset. Evolutionary analyses were conducted in MEGA X [34].

A thorough analysis of the sequenced genome of the ATCC 19606 strain revealed the presence of a filamentous prophage, designated as Af1. Using its key genes, *zot*, *coaA*, and *coaB*, and BLASTn algorithm, the presence of numerous filamentous prophages in *A. baumannii* genomes was detected. The presence of the *zot* gene was detected in 83 of 541 strains, in 15.3% of the total examined strains (Figure 1A). More than half of the strains possessed one *zot* gene—62.2%; two genes were detected in 24.4% and three or more were found in 13.4% of the strains (Figure 1B). Using the amino-acid sequences of the Zot protein, the identified filamentous phages were divided into 10 groups based on sequence similarity, designated by letters from A to J (Figure 1C). The same amino-acid sequence was used to construct a phylogenetic tree, and the results indicated that the prophages were again divided into the same groups (Figure 2).

Using specific primers for the groups, in 23.8% of strains (15/63), the *zot* of Af-related (pro)phages was detected among strains from culture collection (Figure 1C). The A, B and C groups were the most dominant, both among deposited genomes and the *A. baumannii* culture collection.

### 3.2. Genome Organization of Af

**Figure 3:**
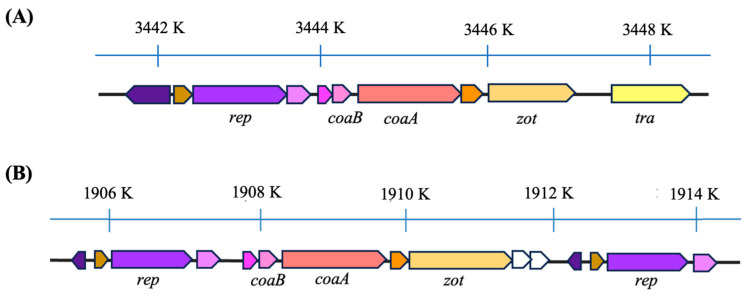
Genome organization of Af phages: (**A**) Af1 prophage in strain ATCC 19606 with transposase and (**B**) a prophage in strain WCHAB005078 (Access. No. CP027246.2; Gorup C), with ends flanked by replicative/regulatory module; *tra*—transposase (yellow); *zot*—gene for Zonula occludens toxin (mustard yellow); *coaA*—gene for minor cot protein (salmon pink); *coaB*—gene for major coat protein (neon pink); *rep*—gene for replication initiation protein (purple); same genes are in same color.

The analysis of Af phage genomes showed that the prophages have key genes specific to viruses belonging to the family *Inoviridae* (Figure 3). The gene configuration bears resemblance to those of other recognized species within the *Inoviridae* family. At one terminus, genes responsible for viral DNA integration (*tra*) into the host genome and virion morphogenesis (*zot*) are present, succeeded by genes responsible for structural proteins (*coaB* and *coaA*).

Based on the comparison of the Af1 prophage genome with the genomes of bacteria that have and do not have this phage integrated, we determined that the most likely coordinates of Af1 phage in strain ATCC 19606 (Access. No. NZ_CP059040.1/CP059040.1) are 3,441,704–3,448,908, so the genome size is 7201 nt. The genome of phage Af1 contains IS110 transposase as a DEDD-type transposase (E = 7.70 × 10^−68^), as well as key genes specific for *Inoviridae*: the gene for zonula occludens toxin (*zot*), with a conserved P-loop-NTP-ase domain (E = 2.01 × 10^−29^); adhesion protein (CoaA), containing a conserved sequence of Neisseria_TspB protein (T and B cell-stimulating protein B; E = 1.18 × 10^−3^); and major coat protein (CoaB), containing 82 amino acids, from which 40 are cleaved as signal peptides (predicted cutting site between 40 and 41aa with a likelihood of 0.7487). The remaining 42 amino acids with conserved CoaB sequences (E = 4.36 × 10^−8^) represent structural proteins with a molecular weight of 4.45 kDa. At the end of the genome is a replication protein, containing a DNA relaxase NicK domain, responsible for recombination, reparation and replication (E = 2.31 × 10^−53^). 

When genomes of prophages from different groups are compared, it is obvious that some possess a transposase, while genomes of some phages are flanked on both sides by a replication/regulation module (Table S3; Figure 3).

Analysis of amino-acid sequences of CoaB and Zot showed <50% similarity within groups from A to J (Table S4) but not among the representatives of the groups, indicating 10 new genera of *A. baumannii* filamentous phages, according to ICTV setup rules for the genus definition [1]. Taking into account two strains that are not included in existing groups and which also have mutually different key genes, the total number of potentially new genera is 12.

### 3.3. Morphology of Af1

**Figure 4:**
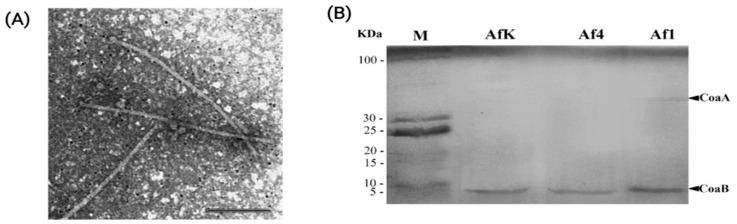
Virion properties of Af phages: (**A**) TEM of Af1 virions obtained by uranyl acetate (1%) contrasting; bar 250 nm; (**B**) Protein profile of AfK, Af4 an Af1 phages; M—LR Unstained protein ladder; in the frame of Af1 phage band, two proteins are indicated: CoaB (approx. 4.5 kDa) and CoaA protein (approx. 49 kDa).

TEM confirmed that Af1 is a complete prophage that produces filamentous virions. Virions were approx. 770 nm long (Figure 4A). Some variations, of shorter or longer virions, were observed in the preparation.

### 3.4. Protein Profile of Af Filamentous Phages

The protein characterization of dialyzed Af filamentous phages using the SDS-PAGE method revealed the presence of major capsid protein (CoaB protein). This was confirmed for all three analyzed phages: Af1, Af4, and AfK (Figure 4B). The size of the bands obtained for the CoaB protein in all three phages corresponds to the expected mass predicted by in silico analysis (approx. 4.5 kDa). An additional protein band, likely the minor coat protein (CoaA), with an approximate molecular weight of 49 kDa, was observed in the Af1 phage. In silico analysis predicted that the molecular mass of CoaA was 48.9 kDa. The CoaA band of Af4 and AfK is not visible, probably due to the low concentration of these proteins.

### 3.5. Af1 Produces Virions with ssDNA

**Figure 5:**
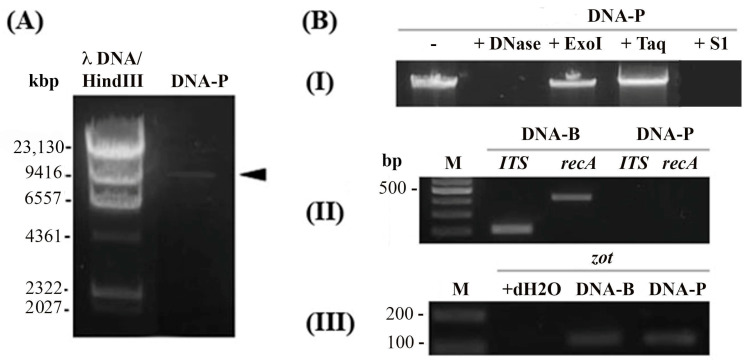
Confirmation of Af1 virion production and circular ssDNA presence in virions. (**A**) Gel electrophoresis of DNA isolated from Af1 virions; Marker λDNA/Hind III; (**B**) Viral DNA digestion by various enzymes: (**I**) Untreated viral DNA as a control; +DNase—treated with DNAse; +ExoI—Exonuclease I treatment; +Taq—treatment with TaqI and +S1—treatment with S1 enzyme; (**II**) PCR using bacterial (DNA-B) and viral DNA (DNA-P) as templates, with primers for *ITS* region and *recA;* M—marker 100 bp; (**III**) PCR using bacterial (DNA-B) and viral DNA (DNA-P) as templates with corresponding primers for *zot* detection; M—marker 100 bp; +dH_2_O—negative control with dH_2_O.

The viral DNA of the Af1 phage was successfully isolated, and the genome size of this phage, according to gel electrophoresis, was between 6557 and 9416 bases (Figure 5A). The precise genome size is impossible to calculate, since dsDNA and ssDNA, as well as linear and circular DNA, do not have the same migration pattern on an agarose gel.

To confirm the type of genome in virions, DNA was treated by various enzymes. The isolated nucleic acid from the virions exhibited sensitivity to DNase, which cleaves both ssDNA and dsDNA, and S1 nuclease (degrading only ssDNA) (Figure 5B-I). However, the band remained uncut after treatment with the TaqI restriction enzyme. This enzyme has 13 cutting sites in Af1 prophage DNA but cannot cut ssDNA. Similarly, no product was obtained using ExoI, which degrades ssDNA with available free ends. The quality of the isolated viral DNA was verified by PCR analysis. The DNA of the Af1 phage did not show the presence of the *ITS* region specific to the *Acinetobacter* genus, nor the *recA* gene specific to *A. baumannii* species (Figure 5B-II), indicating the absence of sample contamination with bacterial DNA. Additionally, the PCR analysis for the presence of the Af1 *zot* gene was successful in both DNA—bacterial and viral (Figure 5B-III).

The same was confirmed for Af4 and AfK.

### 3.6. Plaque Formation Experiments

Neither single- nor double-layer SPOT methods indicated that the phages produce plaques, even in the presence of subinhibitory concentrations of antibiotics 

### 3.7. A. baumannii Infection with Af Phages

**Figure 6:**
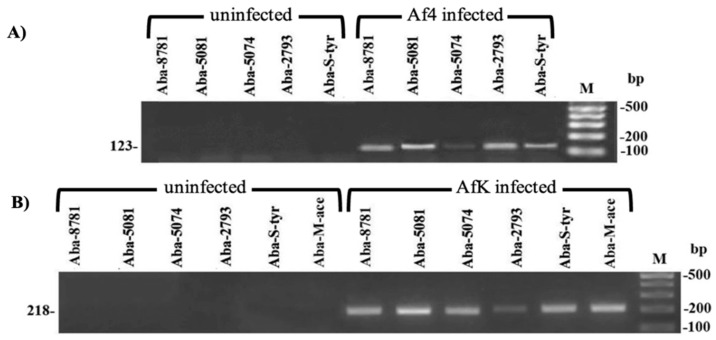
Confirmation of *A. baumannii* strains’ successful infection using Af4 (**A**) and AfK (**B**) specific primer pairs (Table S2); M—marker 100 bp.

The successful infection was observed with the Af4 phage, which belongs to the A group according to designated primers, in five *A. baumannii* strains, while the AfK phage, which belongs to the C group, infected six strains. None of the strains were susceptible to Af1 phage infection. The M-Ace strain was exclusively infected with AfK phage, exhibiting resistance to Af1 and Af4 phages. The infection was confirmed by PCR, using bacterial genomic DNA as a template and corresponding primers (Figure 6), as well as by the sequencing of PCR products (Access. No. OR999321 and OR999322).

### 3.8. The Influence of Afphages on the Host Phenotype

#### 3.8.1. Growth Kinetics

**Figure 7:**
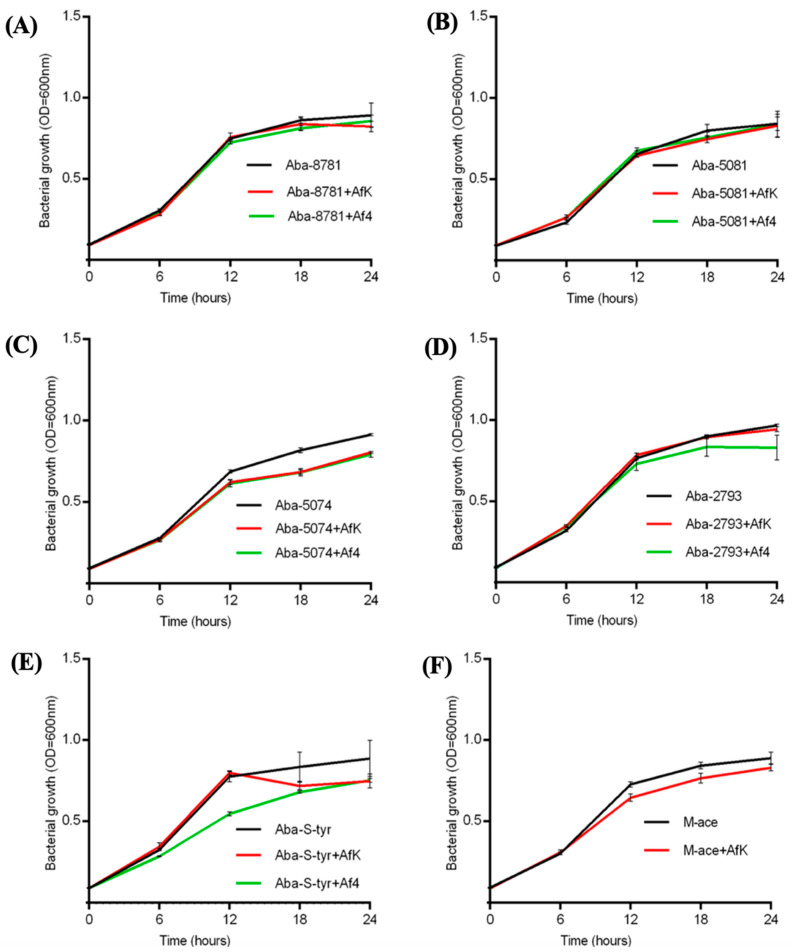
Growth of uninfected, AfK-infected and Af4-infected (except M-ace) *A. baumannii* strains Aba-8781 (**A**), Aba-2793 (**B**), Aba-5074 (**C**), Aba-5081 (**D**), Aba-S-tyr (**E**), and Aba-M-ace (**F**), monitored every 30 min over 24 h and expressed as OD_600_. The results are average + S.D. (*n* = 5).

The strain infection with Af4 and AfK inhibited the growth of *A. baumannii* strains, with exceptions for strains Aba-8781 and Aba-5081 (Figure 7). The changes started after 6–12 h of incubation in comparison to their uninfected counterparts. The infection with Af4 significantly decreased the growth of strains Aba-5074, Aba-2793, Aba-S-tyr and Aba-M-ace, while AfK also decreased the growth of the strains (*p* < 0.001), with the exception of strain Aba-2793.

#### 3.8.2. Twitching Motility

**Figure 8:**
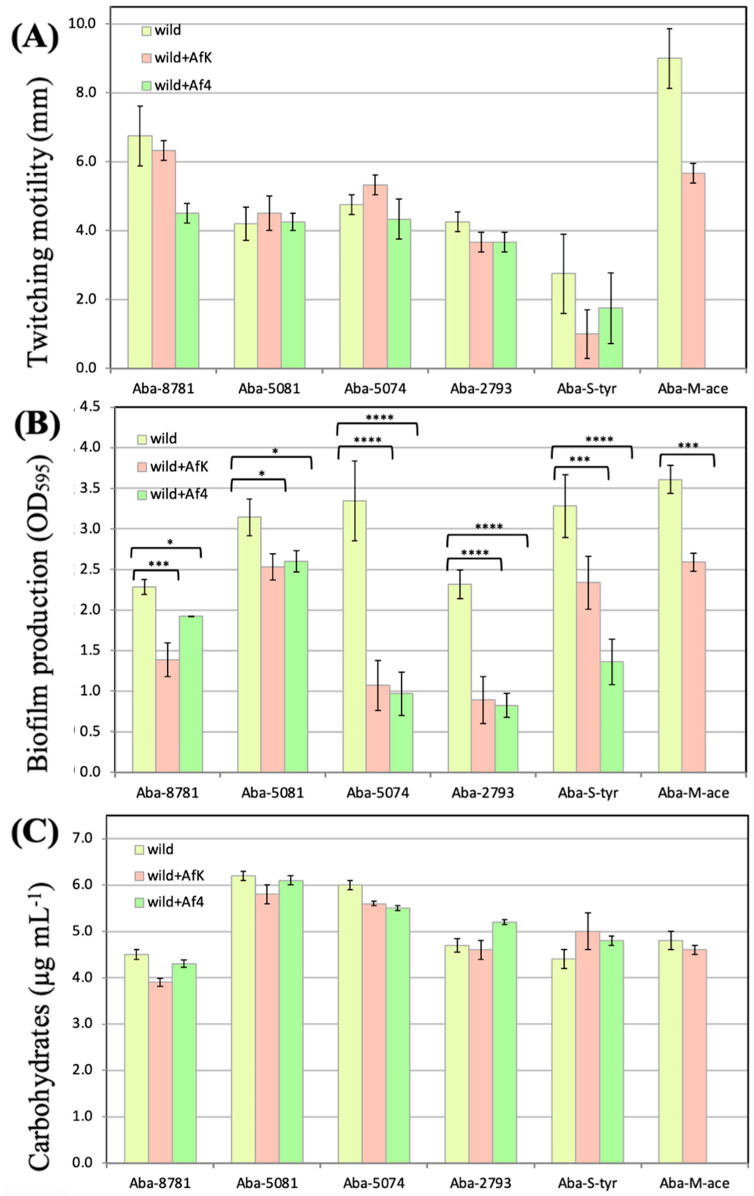
Effect of Af phage infection on *A. baumannii* strain properties: (**A**) Change in twitching motility. The results are average ± SEM (*n* = 4); tested by multiple *t*-tests (GraphPad Prism 6); (**B**) Reduction in biofilm production of *A. baumannii* strain after Af4 and AfK infections, expressed as OD_595_. The results are average ± SEM (*n* = 3); tested by multiple *t*-tests (GraphPad Prism 6); **** *p* < 0.0001; *** *p* < 0.001; * *p* < 0.05. (**C**) Change in carbohydrate production. The results are average ± SEM (*n* = 4); tested by multiple *t*-tests (GraphPad Prism 6).

The twitching motility test indicated slightly inhibited motility in all infected strains, but there was no statistically significant difference between non-infected and infected strains. However, it is important to notice that there was a change from intermediate motile to a twitching-negative group for Aba-8781 infected with the Af4 phage and Aba-5081 infected with the AfK phage (Figure 8A).

#### 3.8.3. Biofilm Formation on Polystyrene Surface

Infections with Af4 and AfK phages significantly contributed to the reduction of biofilm production in various *A. baumannii* strains (Figure 8B). The most significant difference was observed in strains Aba-5074, Aba-2793, and Aba-S-tyr (*p* < 0.0001).

#### 3.8.4. LOS Production

All strains revealed carbohydrate/LOS concentrations in the range from 4 µg mL^−1^ to 8 µg mL^−1^, with no statistically significant difference between uninfected strains and their Af-infected counterparts (Figure 8C).

#### 3.8.5. Antibiotics Susceptibility

Infection with Af4 and AfK phages significantly increased the susceptibility of infected strains to ceftriaxone, tobramycin, and ciprofloxacin (Table 1).

The most obvious change in sensitivity was observed for strain Aba-8781; it was intermediate sensitive to ceftriaxone and resistant to tobramycin, but it was re-sensitized after infection, i.e., it became susceptible to both antimicrobials. Similarly, AfK infection increased the sensitivity of strain Aba-8781 to tobramycin. Strain Aba-5081 exhibited increased susceptibility to ciprofloxacin after infection with both Af4 and AfK phages, although no qualitative changes were observed. Likewise, strain Aba-2793 showed a lower MIC for ceftriaxone after Af phage infection, but it remained resistant to the antibiotic. Finally, the MIC for tobramycin in the Aba-S-tyr strain after Af phage infection decreased four times, but it still remained resistant.

#### 3.8.6. Expression of Efflux Pumps

**Figure 9:**
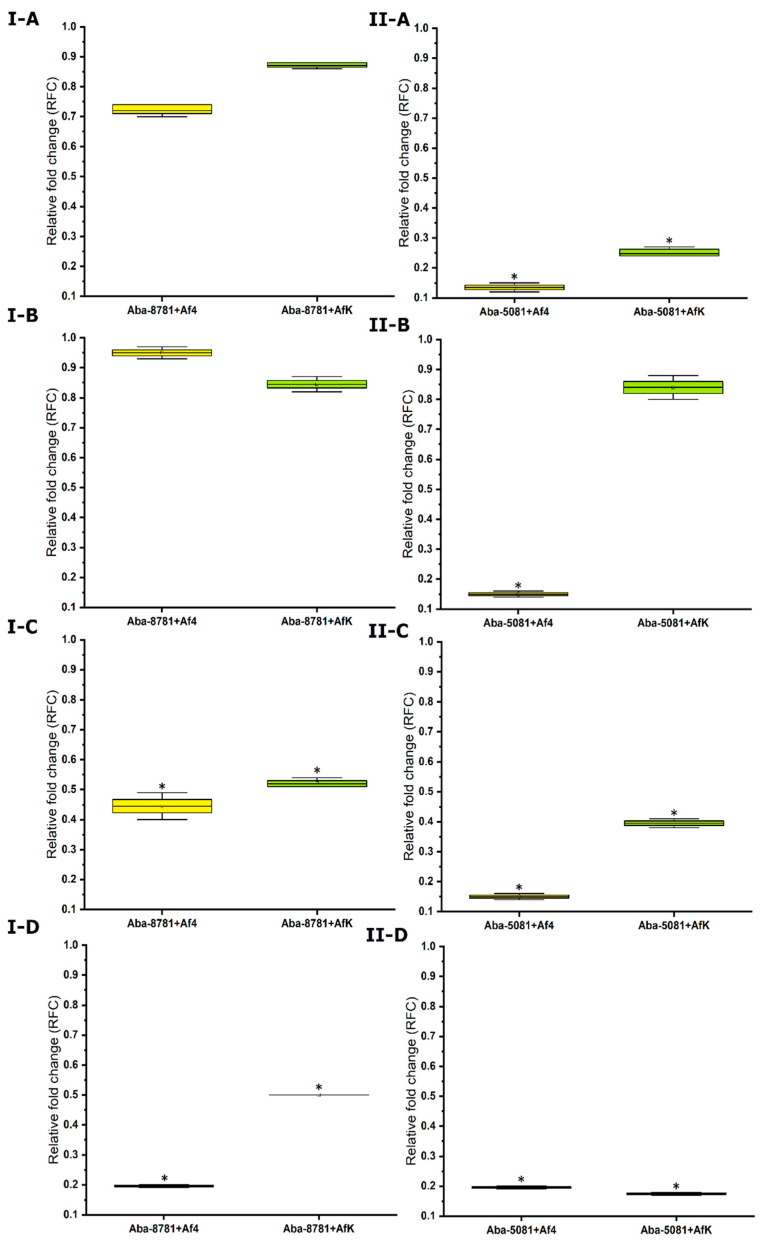
Relative fold change in expression of *adeA* (**A**), *adeB* (**B**), *adeC* (**C**) and *abeMRT* (**D**) efflux pumps in Aba-8781 (**I**) and Aba-5081 (**II**) strains, uninfected and infected with Af4 (yellow) and AfK (green) phages; calculated using 2^−ΔΔCT^ method, and cut-off for relative change in expression is >1.5 or <0.67. * > 1.5 or < 0.67; (*n* = 4).

The relative fold changes of four efflux-pump gene expressions are shown in Figure 9. The downregulation of the genes was detected in each infected strain compared to the uninfected strains. Exceptions were the expression of *adeA* and *adeB* in infected Aba-8781 strain, as well as *adeC* in strain Aba-5081 infected with phage AfK, which are downregulated but significant with regard to a cut-off value of <0.67.

## 4. Discussion

In numerous strains of pathogenic Gram-negative bacteria, filamentous phages from the family *Inoviridae* are widely distributed and occur in plasmid-like or prophage forms. These phages can cause rapid evolution and contribute to the spread of novel genes among their hosts [1]. Therefore, it is very likely that filamentous phages play important roles in the evolution of bacterial species through influencing the ecological adaption and pathogenicity of their hosts. Analysis showed that the examined Af phages of *A*. *baumannii* also belong to the family *Inoviridae* due to their filamentous morphology, specific ssDNA genome organization, virion composition with major coat protein ~5 kDa and adhesion protein of ~50 kDa. Based on the amino-acid sequence of key proteins and recommendations of ICTV, 12 potentially new genera were detected in the frame of the family *Inoviridae*.

Among sequenced genomes of *A. baumannii*, deposited in GenBank, 15.3% of strains possessed at least one *zot* gene, and a slightly higher percentage was detected in the culture collection (23.8%). About two-thirds of Af1-positive strains from GenBank possessed one *zot* gene and thus one prophage, while in the rest, two or more complete or incomplete prophage copies could be found, usually consecutively repeated. Some phages possess a transposase and appear as a single copy in the genome (groups D and I) or as multiple copies (group A). Namely, the Af phage contains IS110 with a DEDD catalytic domain, which does not generate flanking direct repeats upon transposition, and its insertion sequences do not contain inverted repeats. Rather, it uses a “copy-paste” mechanism with a transient double-strand circular DNA intermediate, mediating insertion into repeated extragenic palindromes, integrons, or the ends of other transposable elements [52,53]. Similar multiple prophage copies were previously observed in other prophages that encode their own transposases, such as filamentous phages of *Neisseria*, which encode PivNM/Irg transposase [54], but not in the case of Af phages. Another group of phages, flanked by the gene for replication initiation protein and regulatory genes, probably use host integration machinery and appear as tandem repeats in bacterial genomes. A similar phenomenon was previously observed for Vibrio phage CTXphi and Yersinia phage YpfΦ, which use *dif* sites and host XerCD recombinase, making tandem repeats from two to four prophage copies [55,56,57,58]. During superinfection, the incoming YpfΦ genome inserts itself between two copies of the resident prophage [59], and the same pattern was observed for Af prophages. Besides the multiple copies on one phage, numerous strains were found to contain two or more different prophages, indicating the absence of superinfection exclusion among most Af phage groups.

Among phage proteins, particularly interesting is minor coat protein (CoaA), which is responsible for phage adhesion to bacterial cells. In Af1, it contains a conserved sequence of Neisseria_TspB protein (T and B cell-stimulating protein B; E = 1.18 × 10^−3^), which is recognized as a virulence factor. This protein binds IgG and causes *N. meningitidis* cell aggregation, providing bacteria protection against immune responses [60]. Thus, the CoaA of Af1, due to its similar structure, also may play a role in *A. baumannii* virulence, which should be further elucidated.

We confirmed that virions of the examined Af phages can successfully establish infections of *zot*-negative *A. baumannii* strains. Only Af4 and AfK infections were successful on multiple *A. baumannii* strains, while Af1 did not infect any of the selected strains. While in silico analyses showed the existence of numerous distinct prophages within a single genome, superinfection was not possible in all tested combinations, implying a complex interplay of filamentous bacteriophages within a bacterial host, thus necessitating further investigation into this phenomenon.

*A. baumannii* strains infected with the AfK and Af4 phages showed changes in many phenotypic features when compared to uninfected strains.

Filamentous bacteriophages have been documented to either inhibit bacterial growth [5,7,10,61] or have no significant impact on it [62,63,64]. Similarly, Af filamentous phages were also found to have an effect on bacterial growth upon infection, slightly decreasing it after 6–12 h of incubation. The delayed decrease in growth is probably a consequence of energetic cost and resource consumption during phage replication and viral protein synthesis [65]. Certain filamentous phages are capable of forming plaques on bacterial lawns, which are typically small (~1 mm) and opaque [66]—this is as a result of reduced bacterial growth rather than bacterial lysis. We were unable to demonstrate the presence of Af1, Af4, and AfK plaques on lawns of *A. baumannii*, and this is in accordance with the slight effect of Af phages on *A. baumannii* growth.

Twitching motility, by the utilization of pili type IV, is a common characteristic among *A. baumannii* strains, and at the same time, pili are primary receptors for filamentous phages [1,67]. The infection with Af4 and AfK filamentous phages resulted in a slight inhibition of twitching motility in all infected *A. baumannii* strains. This is consistent with findings for other filamentous phages, suggesting that filamentous phages may influence twitching motility by binding to type IV pili and thereby interfering with the motility and/or decreasing pili production to prevent superinfection [9,10,11,13,68].

It is known that surface carbohydrates, i.e., exopolysaccharide poly-β-(1-6)-N-acetylglucosamine (PNAG), lipooligosaccharide (LOS), and capsular polysaccharides (capsule), affect the fitness and pathogenicity of *A. baumannii*. The capsule presents an important virulence component of *A. baumannii*, allowing resistance to nonspecific and specific host immunity, antibiotic resistance, adhesion, desiccation avoidance, and biofilm production [69,70,71]. In this study, strain ATCC 19606 obtained a carbohydrate amount of 2.84 μg mL^−1^, which corresponds with a previously determined feature that it is nonmucoid and thin-capsulated [48]. A similar pattern of hydrocarbon production was detected in other examined strains. There is no study that has previously examined filamentous phages’ impact on capsule production, and here, we showed that Af prophage has no considerable effect on the quantity of *A. baumannii* carbohydrate production.

Bacteria frequently transition from a planktonic, free-living state to a surface-associated, multicellular lifestyle referred to as a biofilm. The capacity of *A. baumannii* to colonize and form biofilms on both living and non-living surfaces plays a significant role in chronic and persistent infections, antibiotic resistance, as well as its ability to survive in hospital settings and facilitate transmission [72,73]. Although filamentous phages in general contribute to biofilm production, Af phages significantly reduce biofilm mode of *A. baumannii* growth. Similar results were observed only for Ralstonia phages φRSM3 and ϕRSS0 [7,74] or Escherichia phage f1 [75]. The reduction of biofilm production in the case of Af phage infection can be the result of detected bacterial cell lysis absence, so the extracellular DNA as an integral part of the biofilm matrix is released from cells to a lesser extent [76,77]. In addition, filamentous phages of *P. aeruginosa* significantly enhance biofilm stability, being an important component of the biofilm matrix as long filaments (at least 2000 nm) [78]. However, Af phages are three times shorter and probably cannot efficiently bind matrix components like Pf phages. Finally, in the initial steps of biofilm development, pili and twitching motility are reported to be involved in initial attachment and microcolony formation [79], so a slight decrease in twitching motility upon Af phage infection correlates with this finding.

Af filamentous phages contributed to an increased susceptibility of infected strains to specific groups of antibiotics. This was most prominent for tobramycin and ceftriaxone, as Af phage infection re-sensitized bacteria. For instance, phage RSS1 was found to enhance *R. solanacearum* sensitivity to ampicillin by at least tenfold [80]; Pf filamentous bacteriophages increase *P. aeruginosa* sensitivity to aminoglycosides, β-lactams, and fluoroquinolones [13]; and Vibrio phage VEJ increases *V. cholerae* sensitivity to ampicillin [9]. In vitro experiments demonstrated that a combination of filamentous phages and low doses of antibiotics inhibit the growth of or even kill *P. aeruginosa* [81]. The proposed mechanism of antibiotic susceptibility changes has been previously explained by the fact that phage extrusion machinery in the bacterial membrane can change permeability, allowing antibiotic inflow into the cell [13]. In the context of reduced MICs upon infection with filamentous phages, for the first time, we have examined the expression of the efflux pumps in phage-infected strains as a possible mechanism involved in the phenomenon. The efflux-pump AbeMRT in *A. baumannii* strains is responsible for ciprofloxacin resistance [82], so the lower expression in Aba-8781- and Aba-5081-infected strains corresponds to the decreased MICs of ciprofloxacin. Also, the efflux pump AdeABC is associated with resistance to tobramycin and ceftriaxone [81], and its altered expression in infected strains is related to lower MICs. An additional mechanism that may contribute to the functionality of efflux pumps is the potential competition for integration into cell membranes among efflux pumps and extrusion machinery, and/or capsid proteins of Af phages since all of them integrate into cell membranes. A decreased number of efflux pumps integrated into a membrane can result in a smaller amount of eliminated antibiotics from the cell, thus resulting in an increased sensitivity of bacteria, which should be further confirmed.

## 5. Conclusions

The examined prophages of *A. baumannii* share all the common characteristics of the family *Inoviridae*. The data indicate that *A. baumannii*-specific filamentous bacteriophages change bacterial phenotype upon infection, decreasing virulence factors, particularly biofilm production. These findings have fundamental and clinical significance because the presence of these phages in *A. baumannii* strains can indicate the degree of virulence. Taking into account that the infection of *A. baumannii* with these phages increases the bacterial sensitivity to antibiotics, this could be a new strategy for consideration in the context of re-sensitizing pan-resistant strains.

## Figures and Tables

**Table 1:** Changes in antibiotic susceptibility of *A. baumannii* strains after infection with AfK and Af4 phages. Changes ≥ 2 × MIC are in bold; qualitative sensitivity is in parenthesis.

Strain	Ceftriaxone (µg mL^−1^)			Tobramycin (µg mL^−1^)			Ciprofloxacin (µg mL^−1^)		
	Uninfected	+AfK	+Af4	Uninfected	+AfK	+Af4	Uninfected	+AfK	+Af4
Aba-8781	32 (I) *	16 (I)	8 (S)	64 (R)	1 (S)	1 (S)	0.25 (S)	≤0.125 (S)	≤0.125 (S)
Aba-5081	>512 (R)	>512 (R)	512 (R)	64 (R)	32 (R)	64 (R)	128 (R)	32 (R)	32 (R)
Aba-5074	>512 (R)	>512 (R)	>512 (R)	>512 (R)	>512 (R)	>512 (R)	64 (R)	32 (R)	64 (R)
Aba-2793	>512 (R)	128 (R)	128 (R)	1 (S)	1 (S)	1 (S)	0.25 (S)	≤0.125 (S)	≤0.125 (S)
Aba-S-tyr	128 (R)	128 (R)	128 (R)	256 (R)	64 (R)	64 (R)	32 (R)	32 (R)	32 (R)
Aba-M-ace	128 (R)	128 (R)	-	1 (S)	0.5 (S)	-	16 (R)	4 (R)	-
*E. coli* ATCC 25922	0.25 (S)	-	-	0.5 (S)	-	-	<0.125 (S)	-	-

* (R)—resistant; (I) intermediate resistant; (S)—sensitive.

## Data Availability

The original contributions presented in the study are included in the article/Supplementary Materials, further inquiries can be directed to the corresponding author.

## Supplementary Materials

The following supporting information can be downloaded at: https://www.mdpi.com/article/10.3390/v16060857/s1, Table S1: Strains *A. baumannii* deposited in PK Lab culture collection; Table S2: Primers used in the study; Table S3: Presence of Af prophages in genome sequences of *A. baumannii*; Table S4: Matrix tables of prophage genes comparison.
