# Supplementary material for: First Characterization of Acinetobacter baumannii-Specific Filamentous Phages
Source: Viruses. 2024 May 27;16(6):857. doi: 10.3390/v16060857 (PMC11209303; doi:10.3390/v16060857)
Supplement: Supplementary file 1 [file viruses-16-00857-s001.zip › Suppl S1.pdf]

Supplement Table S1. Strains *A.baumannii* deposited in PK Lab culture collection

Strain	Origin	Detected Zot group
ATCC 19606	Reference strain	A
ATCC BAA 747	Reference strain	D, I
NCTC 13423	Reference strain	
8781	Clinical sample- wound infection	
8833	Clinical sample- wound infection	C, D
34963	Clinical sample- wound infection	C
40100	Clinical sample- wound infection	B
2572	Clinical sample- wound infection	B, C
2793	Clinical sample- wound infection	
4156	Clinical sample- wound infection	
4727	Clinical sample- wound infection	E, F
4779	Clinical sample- wound infection	
4803	Clinical sample- wound infection	A, B
4804	Clinical sample- wound infection	A
4890	Clinical sample- wound infection	
4914	Clinical sample- wound infection	C
5055	Clinical sample- wound infection	C, E
5074	Clinical sample- wound infection	
5081	Clinical sample- wound infection	
5372	Clinical sample- wound infection	
6673	Clinical sample- wound infection	
7860	Clinical sample- wound infection	A, B
8255	Clinical sample- wound infection	
B-Fen	Aquatic habitat- surface water	
DP-Fen	Aquatic habitat- surface water	D, H
DN-Ace	Aquatic habitat- surface water	
DZ-Ace	Aquatic habitat- surface water	
M-Ace	Aquatic habitat- surface water	
S-Ace	Aquatic habitat- surface water	E

DTD-Tyr	Aquatic habitat- surface water	
S-Tyr	Aquatic habitat- surface water	
BJ1	Aquatic habitat- surface water	
Aba- M.Bar	Aquatic habitat- surface water	
Aba- G.Pod	Aquatic habitat- surface water	
Aba- Sakajtaš	Aquatic habitat- surface water	
Aba- K	Aquatic habitat- surface water	C
Aba- Karađorđevo	Aquatic habitat- surface water	
Aba- Tam	Aquatic habitat- surface water	
Aba- Sava	Aquatic habitat- surface water	
Aba- Bosut	Aquatic habitat- surface water	
Aba-Vrbas BL	Aquatic habitat- surface water	
Aba-Ar	Aquatic habitat- surface water	
Aba-Jegr	Aquatic habitat- surface water	
Aba-Odž	Waste water	
Aba-Mr.Tisa	Aquatic habitat- surface water	
Aba- Tem	Aquatic habitat- surface water	
Aba- Sirig	Aquatic habitat- surface water	
Aba- Bruje	Aquatic habitat- surface water	
Aba- Beg	Aquatic habitat- surface water	
Aba- N.Miloševo	Aquatic habitat- surface water	
Aba- Karaš	Aquatic habitat- surface water	
Aba- DI	Aquatic habitat- surface water	
Aba- DII	Aquatic habitat- surface water	
Aba- DIII	Aquatic habitat- surface water	
Aba- KanJ	Aquatic habitat- surface water	
Aba- SMil	Waste water	
Aba- Zas	Aquatic habitat- surface water	
Aba- Dub	Aquatic habitat- surface water	
Aba- Vlas	Aquatic habitat- surface water	
Aba- KanBr	Aquatic habitat- surface water	

Aba- KanHip	Aquatic habitat- surface water
Aba- Jama	Waste water
Aba- Rib	Aquatic habitat- surface water
Aba- Ša	Aquatic habitat- surface water
