# Supplementary material for: First Characterization of Acinetobacter baumannii-Specific Filamentous Phages
Source: Viruses. 2024 May 27;16(6):857. doi: 10.3390/v16060857 (PMC11209303; doi:10.3390/v16060857)
Supplement: Supplementary file 1 [file viruses-16-00857-s001.zip › Suppl S2.pdf]

Suppl. Table S2 Primer sused in the study

Target site	Designation	Sequence	Product (bp)	
ITS	P-Ab-ITSF	CATTATCACGGTAATTAGTG	208	Chen et al. (2007)
	P-Ab-ITSR	AGAGCACTGTGCACTTAAG		
recA	P-rA-1	CCTGAATCTTCTGGTAAAAC	425	
	P-rA-2	GTTTCTGGGCTGCCAAACATTAC		
RND Efflux system	adeA For	ATCGCTAACAAAGGCTTGGAA	159	Coyne et al. (2010)
	adeA Rev	CGCCCCCTCAGCTATAGAA		
	adeB For	CTTGCATTTACGTGTGGTGT	168	
	adeB Rev	GCTTTTCTACTGCACCCAAA		
	adeC For	TACACATGCGCATATTGGTG	117	
	adeC Rev	CGTAAAATAACTATCCACTCC		
MATE family Efflux pump	abeMRTF	CCAAAGCAGGTATTGGTCCT	119	
	abeMRTF	CTATTCCGAAGCATTAG		
Housekeeping gene *rpoB*	rpoBF	TCCGCACGTAAAGTAGGAAC	154	Coyne et al. (2010)
	rpoBR	ATGCCGCCTGAAAAAGTAAC		
zot	AAf1-F	GCAGACTTGTAGTAGCTGAACAG	123 bp	This study
	AAf1-R	GGCAAGTGTTTACTACTGGAGAT		
	BAf1-F	CTGGAGTRAGWCCAGCCA	759 bp or 768 bp	
	BAf1-R	TAAGACTGCAATGATGATGGAAMT		
	CAf1-F	TGCTTTACGCATATGYACTTCATC	218 bp	
	CAf1-R	GTCAACCACGTAAYGGCAA		
	DAf1-F	ACATCCGGAGAARACTGGTT	555 bp or 546 bp	
	DAf1-R	TATGTYTGGGCWAACTGTCG		
	EAf1-F	AGCGTCATATTCTTGAGTCTGG	500 bp	
	EAf1-R	AGCATCCGGTTAAGAATGAAAAC		
	FAf1-F	GTGCATTGATAAGGCAGTACCA	417 bp or	
	FAf1-R	GGTTCTGGAAAAACACTTAAAGC		

			420 or 423	
	GAf1-F	TACATCCRSWMAAMACAGG	766 or 793 bp	
	GAf1-R	CCTGAYGATTGGCGWGAR		
	HAf1-F	CATWGAKGTATGWGCYGATGC	281 bp	
	HAf1-R	AAGAGYTWTCRACWCATSGTCAT		
	IAf1-F	AATACGTTGCTGTTCTTCTTGC	602 bp	
	IAf1-R	GCCCWGATGGTTCGATTG		
