# Supplementary material for: First Characterization of Acinetobacter baumannii-Specific Filamentous Phages
Source: Viruses. 2024 May 27;16(6):857. doi: 10.3390/v16060857 (PMC11209303; doi:10.3390/v16060857)
Supplement: Supplementary file 1 [file viruses-16-00857-s001.zip › Suppl S3.pdf]

Suppl. Table S3. Presence of Af prophages in genome sequences of *A. baumannii*

No.	*A. baumannii* strain	Origin	Genome Access. No.	Zot protein designation	*zot* gene coordinates	Zot group	Genome
**1.**	09A16CRGN0014	Human isolate	NZ_CP034242.1/CP034242.1				
**2.**	09A16CRGN003B	Nosocomial infection	NZ_CP034243.1/CP034243.1				
**3.**	10_3	Patient	NZ_CP059547.1/CP059547.1	UAB24888.1	1,863,174..1,864,454	H	Flanked[1]
**4.**	10_4	Field soil	NZ_CP059546.1/CP059546.1	UAB21455.1<br>UAB21447.1	1,869,334..1,870,614<br>1,863,204..1,864,484	H<br>H	flanked<br>flanked
**5.**	10042	Secretion	NZ_CP023026.1/CP023026.1				
**6.**	10324	Bronchial fluid	NZ_CP023022.1/CP023022.1				
**7.**	11A1213CRGN008	Nosocomial infection	NZ_CP035186.1/CP035186.1				
**8.**	11A1213CRGN055	Nosocomial infection	NZ_CP035185.1/CP035185.1				
**9.**	11A1213CRGN064	Nosocomial infection	NZ_CP043419.1/CP043419.1				
**10.**	11A1314CRGN088	Nosocomial infection	NZ_CP035184.1/CP035184.1				
**11.**	11A1314CRGN089	Nosocomial infection	NZ_CP043418.1/CP043418.1				
**12.**	11A14CRGN003	Nosocomial infection	NZ_CP035183.1/CP035183.1				
**13.**	11W359501	Human isolate	CP041035.1	QDE16876.1	2,054,177..2,055,352<br>2074,055..2,075,300<br>2,062,895..2,064,174	E<br>pseudo<br>pseudo	flanked
**14.**	1326359	Environment	CP107577.1				
**15.**	1326525-1	Skin	CP107579.1				
**16.**	1326525-2	Skin	CP107581.1				
**17.**	1326525-3	Skin	CP107583.1				
**18.**	1326527-1	Skin	CP107585.1				
**19.**	1326527-2	Skin	CP107587.1				
**20.**	1326569	Skin	CP107590.1				
**21.**	1326580	Skin	CP107593.1				
**22.**	1326581-1	Wound	CP107595.1				
**23.**	1326581-2	Wound	CP107597.1				
**24.**	1326584	Skin	CP107599.1				
**25.**	1326589	Skin	CP107601.1				
**26.**	1326595	Skin	CP107603.1				
**27.**	1326924-1	Environment	CP107605.1				
**28.**	1326924-2	Environment	CP107608.1				
**29.**	1326924-3	Environment	CP107610.1				
**30.**	1326927-1	Environment	CP107612.1				
**31.**	1326927-2	Environment	CP107614.1				
**32.**	1326932	Environment	CP107616.1				
**33.**	15A34	Pulmonary	NZ_CP020590.1/CP020590.1				
**34.**	15A5	Pulmonary	NZ_CP020574.1/CP020574.1				

**35.**	1656-2	Clinicalisolates	NC_017162.1/CP001921.1				
**36.**	17-84	Human	NZ_CP059479.1/CP059479.1	QNB02254.1	2,631,785..2,632,945	F	Classic[2]
**37.**	2008S11-069	Clinicalisolates	CP033516.1				
**38.**	2014BJAB1	Blood	NZ_CP059354.1/CP059354.1				
**39.**	2014LNAB1	Blood	NZ_CP059352.1/CP059352.1				
**40.**	2014TJAB1	Blood	NZ_CP059349.1/CP059349.1				
**41.**	2016BJAB1	Sputum	NZ_CP059355.1/CP059355.1				
**42.**	2016GDAB1	Bronchoalveolar lavage fluid	NZ_CP065051.1/CP065051.1				
**43.**	2016LNAB1	Cerebrospinal fluid	NZ_CP059353.1/CP059353.1				
**44.**	2018BJAB1	Sputum	NZ_CP059351.1/CP059351.1				
**45.**	2018BJAB2	Bronchial	NZ_CP059350.1/CP059350.1				
**46.**	2018HBAB1	Abdominal fluid	NZ_CP059356.1/CP059356.1				
**47.**	2018HLJAB1	Pus	NZ_CP059358.1/CP059358.1				
**48.**	2018HLJAB2	Abdominal fluid	NZ_CP059357.1/CP059357.1				
**49.**	2018TJAB1	Blood	NZ_CP059359.1/CP059359.1				
**50.**	2021CK-01300	Woundabscess	NZ_CP104342.1/CP104342.1				
**51.**	2021CK-01332	Wound/abscess	NZ_CP104347.1/CP104347.1				
**52.**	2021CK-01333	Sputum	NZ_CP104350.1/CP104350.1				
**53.**	2021CK-01335	Wound/abscess	NZ_CP104351.1/CP104351.1				
**54.**	2021CK-01407	Sputum	NZ_CP104449.1/CP104449.1				
**55.**	2021CK-01408	Blood	NZ_CP104335.1/CP104335.1				
**56.**	2021CK-01409	Wound/abscess	NZ_CP104340.1/CP104340.1				
**57.**	2022CK-00337	Sputum	CP115629.1				
**58.**	2022CK-00839	Blood	CP117762.1				
**59.**	280820	Blood	NZ_CP098791.1/CP098791.1	USI39205.1<br>USI37014.1<br>USI37022.1<br>USI37030.1	487,300..488,364<br>2,108,991..2,110,055<br>2,114,690..2,115,754<br>2,122,564-2,123,442	A<br>A<br>A<br>I	transposase[3]<br>classic<br>classic<br>transposase
**60.**	29FS20	Feces	CP044519.1/CP044519.1				
**61.**	31FS3-2	Feces	NZ_CP044517.1/CP044517.1	QLF06782.1	1,984,690..1,985,904	G	flanked
**62.**	3207	Bronchial fluid	CP015364.1				
**63.**	36-1512	Respiratory tract	NZ_CP059386.1/CP059386.1				
**64.**	37662	Sputum	NZ_CP104751.1/CP104751.1				
	37662RM1	Sputum	CP116803.1				
	37662RM2	Sputum	NZ_CP116801.1/CP116801.1				
**65.**	40288	Urine	NZ_CP077801.1/CP077801.1				
**66.**	5388	Blood	NZ_CP096768.1/CP096768.1				
**67.**	5626	Sputum	NZ_CP096766.1/CP096766.1				
**68.**	5634	Sputum	NZ_CP096764.1/CP096764.1				
**69.**	5651	Sputum	NZ_CP096762.1/CP096762.1				
**70.**	5653	Sputum	NZ_CP096759.1/CP096759.1				
**71.**	5656	Blood	NZ_CP096757.1/CP096757.1				

**72.**	5663	Blood	NZ_CP096755.1/CP096755.1				
**73.**	5664	Blood	NZ_CP096753.1/CP096753.1				
**74.**	5666	Blood	NZ_CP096749.1/CP096749.1				
**75.**	5669	Blood	NZ_CP096747.1/CP096747.1				
**76.**	5670	Blood	NZ_CP096745.1/CP096745.1				
**77.**	5671	Blood	NZ_CP096742.1/CP096742.1				
**78.**	5672	Blood	NZ_CP096740.1/CP096740.1				
**79.**	5679	Blood	NZ_CP096738.1/CP096738.1				
**80.**	5683	Blood	NZ_CP096735.1/CP096735.1				
**81.**	5685	Blood	NZ_CP096734.1/CP096734.1				
**82.**	5689	Blood	NZ_CP096731.1/CP096731.1				
**83.**	5729	Secretion	NZ_CP096729.1/CP096729.1				
**84.**	5732	Sputum	NZ_CP096727.1/CP096727.1				
**85.**	5734	Blood	NZ_CP096724.1/CP096724.1				
**86.**	5736	Sputum	NZ_CP096722.1/CP096722.1				
**87.**	5740	Sputum	NZ_CP096720.1/CP096720.1				
**88.**	5741	Sputum	NZ_CP096717.1/CP096717.1				
**89.**	5745	Abdominal fluid	NZ_CP096714.1/CP096714.1				
**90.**	5759	Bronchoalveolar lavage fluid	NZ_CP096710.1/CP096710.1				
**91.**	5760	Sputum	NZ_CP096707.1/CP096707.1				
**92.**	5761	Blood	NZ_CP096705.1/CP096705.1				
**93.**	5765	Blood	NZ_CP096704.1/CP096704.1				
**94.**	5767	Blood	NZ_CP096702.1/CP096702.1				
**95.**	5768	Blood	NZ_CP096700.1/CP096700.1				
**96.**	5769	Bronchial	NZ_CP096698.1/CP096698.1				
**97.**	5771	Bronchoalveolar lavage fluid	NZ_CP096696.1/CP096696.1				
**98.**	5773	Secretion	NZ_CP096693.1/CP096693.1				
**99.**	5779	Sputum	NZ_CP096692.1/CP096692.1				
**100.**	5839	Blood	NZ_CP096690.1/CP096690.1				
**101.**	5840	Blood	NZ_CP096688.1/CP096688.1				
**102.**	5845	Wound	NZ_CP023034.1/CP023034.1				
**103.**	5846	Blood	NZ_CP096686.1/CP096686.1				
**104.**	5847	Sputum	NZ_CP096684.1/CP096684.1				
**105.**	5955	Tracheal aspirate	NZ_CP096682.1/CP096682.1				
**106.**	6080	Blood	NZ_CP096681.1/CP096681.1	UQL19311.1	1,757,214..1,758,470	I	classic
**107.**	6200	Bodily fluid	CP010397.1	AJB68401.1	3,486,034..3,487,509	D	transposase
**108.**	6507	Human isolate	NZ_CP045528.1/CP045528.1				
**109.**	7804	Bronchoalveolar lavage fluid	CP022283.1	ASO71342.1	2,358,478..2,359,542	A	flanked
**110.**	7835	Human tissue	NZ_CP033243.1/CP033243.1				
**111.**	7847	Blood	NZ_CP023031.1/CP023031.1				
**112.**	9102	Bronchial fluid	NZ_CP023029.1/CP023029.1				
**113.**	810CP	Feces	NZ_CP026338.1/CP026338.1	AXG85036.1	2,013,496..2,014,710	B	flanked

**114.**	9201	Blood	NZ_CP023020.1/CP023020.1				
**115.**	A1	Human clinical	NZ_CP010781.1/CP010781.1				
**116.**	A118	Bloodculture	NZ_CP059039.1/CP059039.1				
		A118 DeltaH0N27_10825::kan	CP113070.1				
		A118 DeltaH0N27_10830::kan	CP113071.1				
		A118 DeltaH0N27_10820::kan H0N27_10830-K176N	CP113073.1				
		A118 DeltaH0N27_10820-30::kan	CP113072.1				
**117.**	A1296	Sputum	NZ_CP018332.1/CP018332.1	ATI37470.1	457,231..458,295	A	transposase
**118.**	A1429	Secretion	NZ_CP046898.1/CP046898.1	QLB36172.1	2,720,803..2,722,053	I	classical
**119.**	A320 (RUH134)	Reference strain for global clone 2	NZ_CP032055.1/CP032055.1				
**120.**	A388	Variant ofglobal clone 1	NZ_CP024418.1/CP024418.1	ATP87138.1<br>ATP87121.1	1,988,818..1,989,882<br>1,978,284..1,979,528	A<br>C	flanked<br>flanked
**121.**	A52	Sputum	NZ_CP034092.1/CP034092.1		-		
**122.**	A54	A54r	NZ_CP099857.1/CP099857.1				
		A54s	NZ_CP099858.1/CP099858.1				
**123.**	A85	Sputum	CP021782.1	ASF77351.1<br>ASF77364.1	2,089,961..2,091,127<br>2,098,290..2,099,465	C<br>E	flanked<br>flanked
**124.**	Ab-3556	Clinical isolate	NZ_CP104786.1/CP104786.1				
**125.**	Ab-3557	Clinical isolate	NZ_CP104907.1/CP104907.1				
**126.**	Ab-B004d-c	Sputum	CP051875.1	QJF35496.1<br>QJF35489.1	2,019,580..2,020,803<br>2,014,411..2,015,595	B<br>F	flanked<br>classical
**127.**	Ab-C102	Blood	NZ_CP051862.1/CP051862.1				
**128.**	Ab-C63	Sputum	CP051866.1	QJF39179.1	1,866,099..1,867,313	B	flanked
**129.**	Ab-D10a-a	Cerebrospinal fluid	CP051869.1	 QJF32742.1<br>QJF32748.1	3,344,073..3,345,298<br>3,350,699..3,351,952<br>3,355,906..3,357,090	Pseudo<br>B<br>F	 incomplete<br>classical
**130.**	AB0057	Blood stream isolate	CP001182.2	ACJ41387.1<br>ACJ41400.1<br>ACJ41408.1<br>ACJ41419.1<br>ACJ41435.1<br>ACJ41441.1	2,107,602..2,108,768<br>2,116,084..2,117,250<br>2,122,252..2,123,427<br>2,130,964..2,132,130<br>2,141,666..2,142,832<br>2,145,686..2,146,930	C<br>C<br>E<br>C<br>C<br>C	flanked<br>classical<br>flanked<br>flanked<br>flanked<br>classical
**131.**	AB030	Hospital	CP009257.1	AIL80789.1	3,918,472..3,919,686	B	flanked
**132.**	AB031	Hospital	CP009256.1	AIL75680.1	2,291,172..2,292,347	E	flanked
**133.**	Ab04-mff	Blood	NZ_CP012006.1/CP012006.1				
**134.**	AB043	Generated in lab from ATCC 17979	NZ_CP043910.1/CP043910.1				
**135.**	AB105	Unknown	NZ_CP103338.1/CP103338.1	UWZ62185.1	388,749..390,212	D	transposase
**136.**	AB14-VUB	Clinical isolates	NZ_CP091376.1/CP091376.1				

**137.**	AB16-VUB	Clinical isolates	NZ_CP091375.1/CP091375.1				
**138.**	AB167-VUB	Clinical isolates	NZ_CP091368.1/CP091368.1				
**139.**	AB169-VUB	Clinical isolates	NZ_CP091367.1/CP091367.1	UMN21410.1<br>UMN21420.1<br>UMN21397.1	997,270..998,334<br>1,005,254..1,006,429<br>988,551..989,270<br>1,011,375..1,012,551	A<br>A<br>E<br>pseudo	flanked<br>flanked<br>flanked
**140.**	AB171-VUB	Clinical isolates	NZ_CP091366.1/CP091366.1				
**141.**	AB172-VUB	Clinical isolates	NZ_CP091365.1/CP091365.1				
**142.**	AB173-VUB	Clinical isolates	NZ_CP091364.1/CP091364.1				
**143.**	AB175-VUB	Clinical isolates	NZ_CP091363.1/CP091363.1				
**144.**	AB176-VUB	Clinical isolates	NZ_CP091362.1/CP091362.1				
**145.**	AB177-VUB	Clinical isolates	NZ_CP091361.1/CP091361.1	UMN42652.1	406,847..407,911	A	flanked
**146.**	AB179-VUB	Sputum	NZ_CP091360.1/CP091360.1				
**147.**	AB180-VUB	Clinical isolate	NZ_CP091359.1/CP091359.1				
**148.**	AB181-VUB	Clinical isolate	NZ_CP091358.1/CP091358.1				
**149.**	AB183-VUB	Clinical isolate	NZ_CP091357.1/CP091357.1				
**150.**	AB186-VUB	Clinical isolate	NZ_CP091356.1/CP091356.1	UMN60612.1	3,567,519..3,568,583	A	Flanked (pseudogenes)
**151.**	AB187-VUB	Clinical isolate	NZ_CP091355.1/CP091355.1				
**152.**	AB188-VUB	Clinical isolate	NZ_CP091354.1/CP091354.1				
**153.**	AB189-VUB	Clinical isolate	NZ_CP091353.1/CP091353.1				
**154.**	AB193-VUB	Clinical isolate	NZ_CP091352.1/CP091352.1				
**155.**	AB194-VUB	Clinical isolate	NZ_CP091351.1/CP091351.1				
**156.**	AB20-VUB	Clinical isolate	NZ_CP091374.1/CP091374.1				
**157.**	AB21-VUB	Clinical isolate	NZ_CP091373.1/CP091373.1				
**158.**	AB212-VUB	Clinical isolate	NZ_CP091350.1/CP091350.1				
**159.**	AB213-VUB	Clinical isolate	NZ_CP091349.1/CP091349.1				
**160.**	AB214-VUB	Clinical isolate	NZ_CP091348.1/CP091348.1				
**161.**	AB216-VUB	Clinical isolate	NZ_CP091347.1/CP091347.1				
**162.**	AB217-VUB	Clinical isolate	NZ_CP091346.1/CP091346.1				
**163.**	AB219-VUB	Clinical isolate	NZ_CP091345.1/CP091345.1				
**164.**	AB220-VUB	Clinical isolate	NZ_CP091344.1/CP091344.1				
**165.**	AB222-VUB	Clinical isolate	NZ_CP091343.1/CP091343.1				
**166.**	AB224-VUB	Clinical isolate	NZ_CP091342.1/CP091342.1				
**167.**	AB226-VUB	Clinical isolate	NZ_CP091341.1/CP091341.1				
**168.**	AB227-VUB	Clinical isolate	NZ_CP091340.1/CP091340.1				
**169.**	AB229-VUB	Clinical isolate	NZ_CP091339.1/CP091339.1				
**170.**	AB231-VUB	Clinical isolate	NZ_CP091338.1/CP091338.1				
**171.**	AB232-VUB	Clinical isolate	NZ_CP091337.1/CP091337.1				
**172.**	AB2369	Sputum	NZ_CP103413.1/CP103413.1				
**173.**	AB2877	Sputum	NZ_CP092485.1/CP092485.1				
**174.**	AB3-VUB	Clinical isolate	NZ_CP091378.1/CP091378.1				

**175.**	AB307-0294	Human blood	NZ_CP001172.1/CP001172.2		-		
**176.**	AB32-VUB	Clinical isolate	NZ_CP091372.1/CP091372.1				
**177.**	AB322	Blood	CP119232.1	WEI07792.1	1,374,211..1,375,455	C	flanked
**178.**	AB329	Sputum	NZ_CP091452.1/CP091452.1				
**179.**	AB34299	Human isolate	CP014291.1		-		
**180.**	AB36-VUB	Clinical isolate	NZ_CP091371.1/CP091371.1				
**181.**	AB39-VUB	Clinical isolate	NZ_CP091370.1/CP091370.1				
**182.**	AB3927	Bronchoalveolar lavage fluid	NZ_CP102831.1/CP102831.1				
**183.**	AB40-VUB	Clinical isolates	NZ_CP091369.1/CP091369.1				
**184.**	AB43	Sputum	NZ_CP083181.1/CP083181.1	UAS49025.1	1,923,886..1,925,052	C	flanked
**185.**	AB44	Sputm	CP107728.1				
**186.**	AB4451	Sputum	NZ_CP095091.1/CP095091.1				
**187.**	AB4568	Sputum	NZ_CP024613.1/CP024613.1				
**188.**	AB4653	Sputum	NZ_CP024612.1/CP024612.1				
**189.**	Ab4977	Sputum	NZ_CP024611.1/CP024611.1				
**190.**	AB5075-UW	Tibia/osteomyelitis	NZ_CP008706.1/CP008706.1	AKA31722.1<br>AKA31743.1<br>AKA31754.1<br>AKA31733.1	1,994,157..1,995,317<br>1,999,414..2,000,658<br>2,005,890..2,007,134<br>2,012,367..2,013,611	F<br>C<br>C<br>C	classical<br>classical<br>classical<br>flanked
**191.**	AB5075-VUB	Osteomyelitis	NZ_CP070362.2/CP070362.2	UEL55533.1<br>UEL55536.1<br>UEL55541.1<br>QRY90003.1<br>QRY89980.1<br>QRY89988.1<br>QRY89996.1	1,508,352..1,509,515<br>1,516,058..1,517,026<br>1,530,889..1,532,049<br>1,555,568..1,556,812<br>1,536,145..1,537,389<br>1,542,617..1,543,861<br>1,549,092..1,550,336	F<br>F<br>F<br>C<br>C<br>C<br>C	flanked<br>classical<br>classical<br>classical<br>classical<br>classical<br>flanked
**192.**	AB5075-VUB-itrA::ISAba13	Clinical isolate	NZ_CP070358.1/CP070358.2				
**193.**	AB5116	Sputum	NZ_CP091173.1/CP091173.1				
**194.**	AB6870155	Human sputum	NZ_CP114381.1/CP114381.1	WAU38677.1<br>WAU38687.1<br>WAU38701.1<br>WAU38715.1	1,959,681-1,960,847<br>1,965,939..1,967,105<br>1,975,300..1,976,475<br>1,984,219..1,985,385	C<br>C<br>E<br>C	classical<br>flanked<br>flanked<br>flanked
**195.**	ab736	Human isolate	CP015121.1	ARN32238.1	3,446,367..3,447,482	A	transposase
**196.**	AB79	Eyesecretion	NZ_CP054416.1/CP054416.1				
**197.**	AB9-VUB	Clinical isolate	NZ_CP091377.1/CP091377.1				
**198.**	Aba	Clinical isolate	NZ_CP030083.1/CP030083.1				
**199.**	abA1	Bronchial	NZ_CP060732.1/CP060732.1				
**200.**	AbCAN2	Coccygeal isolate	NZ_CP045428.1/CP045428.1				
**201.**	ABCR01	Sputum	NZ_CP042931.1/CP042931.1				
**202.**	AbCTX1	Throat sample	NZ_CP060029.1/CP060029.1		-		
**203.**	AbCTX13	Peritoneal fluid	NZ_CP059729.1/CP059729.1				
**204.**	AbCTX5	Catheter	NZ_CP060505.1/CP060505.1				

**205.**	AbCTX9	Bronchoalveolar lavage	NZ_CP060504.1/CP060504.1				
**206.**	ABF9692	Trachea	CP048827.1	QJP36502.1<br>QJP37779.1	548,202..549,266<br>2,024,695..2,025,947	A<br>I	transposase<br>classical
**207.**	AbH12O-A2	Nosocomial infection	NZ_CP009534.1/CP009534.1	AIS06532.1<br>AIS06539.1	1,918,721..1,919,944<br>1,924,506..1,925,666	B<br>F	flanked<br>classical
**208.**	ABNIH28	Hospital environment	NZ_CP026125.1/CP026125.1				
**209.**	AbPK1	Sheep broncho-alveolar lavage	CP024576.1/CP024576.1				
**210.**	ABUH763	Human isolate	NZ_CP035051.1/CP035051.1				
**211.**	ABUH773	Human isolate	NZ_CP035049.1/CP035049.1				
**212.**	ABUH793	Human isolate	NZ_CP035045.1/CP035045.1				
**213.**	ABUH796	Human isolate	NZ_CP035043.1/CP035043.1				
**214.**	AC1633	Blood	CP059300.1	QOJ60985.1<br>QOJ58826.1<br>QOJ60978.1	1,043,382..1,044,446<br>2,873,057..2,874,121<br>1,036,784..1,038,037	A<br>A<br>I	flanked<br>transposase<br>flanked
**215.**	AC29	Endotracheal secretion	NZ_CP007535.2/CP007535.2				
**216.**	AC30	Endotrachial secretion	CP007577.1		-		
**217.**	ACI713	Human isolate	NZ_CP086759.1/CP086759.1				
**218.**	ACICU	Cerebrospinal fluid	NC_010611.1/CP000863.1		-		
			NC_010611.1/CP000863.1				
**219.**	ACN21	Blood	CP038644.1	QBY88097.1	125,355..126,419	A	flanked
**220.**	AF-401	Small colon	CP018254.1				
**221.**	AF-673	Sputum	NZ_CP018256.1/CP018256.1				
**222.**	AOR07-BL	Blood	NZ_CP102762.1/CP102762.1				
**223.**	AR_0052	Unknown	CP027183.1	AVI37409.1	1,489,528..1,490,751	B	flanked
**224.**	AR_0056	Unknown	NZ_CP026707.1/CP026707.1<br>NZ_CP027123.1/CP027123.1				
**225.**	AR_0056	Unknown	NZ_CP027123.1/CP027123.1<br>NZ_CP026707.1/CP026707.1				
**226.**	AR_0063	Unknown	CP026711.1				
**227.**	AR_0070	Unknown	CP027178.1	AVI31830.1	1,805,197..1,806,420	B	flanked
**228.**	AR_0078	Unknown	NZ_CP026761.1/CP026761.1				
**229.**	AR_0083	Unknown	CP027528.1	AVN24918.1	944,082..945,248	C	flanked
**230.**	AR_0088	Unknown	NZ_CP027530.1/CP027530.1				
**231.**	AR_0101	Unknown	CP027611.1				
**232.**	AR_0102	Unknown	NZ_CP027607.1/CP027607.1				
**233.**	ATCC 17961	Blood	CP065432.1	QPP13769.1	3,474,160..3,475,275	A	transposase
**234.**	ATCC 17978	Urine	NZ_CP099855.1/CP099855.1	USY45590.1	1,930,215..1,931,429	B	flanked
			NZ_CP099856.1/CP099856.1	USY42031.1	1,940,608..1,941,822		
			CP033108.1	UZK34645.1	1,940,550..1,941,764		
			CP033110.1	UZK38384.1	1,940,548..1,941,762		
			CP000521.1				

			NZ_CP053098.1/CP053098.1				
			NZ_CP049363.1/CP049363.1				
			NZ_CP059041.1/CP059041.1				
			NZ_CP053098.1/CP053098.1				
			NZ_CP074710.1/CP074710.1				
		Tnarac-H0N27_12600	CP113074.1				
		ATCC 17978-mff	NZ_CP012004.1/CP012004.1				
		Atcc 17978-vu	NZ_CP018664.1/CP018664.1				
		SubstrainPmr-low	NZ_CP039023.2/CP039023.2				
		SubstrPmr-high	NZ_CP039025.2/CP039025.2				
		17978UN	NZ_CP079931.1/CP079931.1	QXZ49850.1	1,940,625..1,941,839		
		ATCC 17978-VUB	NZ_CP091335.1/CP091335.1	UMO36571.1	711,730..712,944		
		AB042 lab mutation of ATCC 17978	NZ_CP019034.1/CP019034.1				
**235.**	ATCC BAA1605	Sputum	CP058625.1	QLG82246.1	376..1,621<br>4,022,320..4,023,495<br>4,028,494..4,029,659	pseudo<br>E<br>pseudo	flanked
**236.**	ATCC BAA-1790	Sputum	CP042841.1	QEE57420.1	2,052,806..2,054,020	B	flanked
**237.**	ATCC 19606	Urine	NZ_CP059040.1/CP059040.1	QNT84578.1	3,446,331..3,447,446	A	transposase
			NZ_CP046654.1/CP046654.1	QGX50140.1	1,854,468..1,855,583		
			NZ_CP045110.1/CP045110.1	QFQ03982.1	551,050..552,165		
			NZ_CP074585.1/CP074585.1	WP_000585018.1	647,895..649,010		
		ATCC19606-VUB	NZ_CP091334.1/CP091334.1	UMO38739.1	3,021,399..3,022,514		
		CIP 70.34=JCM 6841	NZ_CP058289.1/CP058289.1	QXV70862.1	3,166,544..3,167,659		
		PartI-Abaumannii-RM8376ATCC isolate	NZ_CP064375.1/CP064375.1	UON33098.1	1,666,796..1,667,911		
**238.**	Ax270	Cat	NZ_CP049240.1/CP049240.1				
**239.**	AYE	Pneumonia and urinary tract	CU459141.1	CAM86718.1<br>CAM86731.1	1,901,138..1,902,469<br>1,909,745..1,911,076	C<br>C	flanked<br>flanked
**240.**	AYP-A2	Wound	NZ_CP024124.1/CP024124.1				
**241.**	B10	Sputum	NZ_CP079942.1/CP079942.1				
**242.**	B8300	Blood	CP021347.1	KMV25700.1	1,114,131..1,115,591	D	transposase
**243.**	B8342	Blood	CP021342.1	KMV08265.1<br>KMV08135.1	1,933,645..1,935,132<br>3,573,316..3,574,482	D<br>C	transposase<br>flanked
**244.**	B9	Seine river	NZ_CP059548.1/CP059548.1				
**245.**	BAL062	Unknown	NZ_LT594095.1/LT594095.1				
**246.**	BJAB07104	Clinical	NC_021726.1/CP003846.1		-		
**247.**	BJAB0715	Clinical	NC_021733.1/CP003847.1	AGQ06650.1	2,098,155..2,099,225	F	incomplete
**248.**	BJAB0868	Clinical	NC_021729.1/CP003849.1		-		
**249.**	BM2333	Bloodstream infections	NZ_CP091328.1/CP091328.1				
**250.**	C25	Cerebral-spinal fluid	NZ_CP032743.1/CP032743.1				

**251.**	C9	Draining-matting conveyor	NZ_CP113442.1/CP113442.1				
**252.**	CA-17	Hunam isolate	NZ_CP012587.1/CP012587.1				
**253.**	CAb-65	Fecal	NZ_CP060994.1/CP060994.1				
**254.**	CAM180-1	Oral cavity	NZ_CP044356.1/CP044356.1				
**255.**	CBA7	Sputum	NZ_CP020586.1/CP020586.1				
**256.**	CCBH31258	Catheter tip	CP101889.1				
**257.**	CFSAN093705	Urine	NZ_CP061525.1/CP061525.1				
**258.**	CFSAN093706	Blood	NZ_CP061523.1/CP061523.1				
**259.**	CFSAN093707	Sterile body site	NZ_CP061522.1				
**260.**	CFSAN093708	Wound	NZ_CP061519.1/CP061519.1				
**261.**	CFSAN093709	Urine	NZ_CP061517.1/CP061517.1				
**262.**	CFSAN093710	Sterile body site	NZ_CP061514.1/CP061514.1				
**263.**	CI300	Tracheal aspiray	NZ_CP082952.1/CP082952.1	UBK06240.1	2,058,126..2,059,190	A	flanked
**264.**	Cl415	Blood	NZ_CP071763.1/CP071763.1				
**265.**	CIAT758	Blood	NZ_CP038500.1/CP038500.1				
**266.**	CIP70.10	Human skin tissue	LN865143.1	CRL93204.1<br>CRL94564.1	491,945..493,009<br>1,965,833-1,967,086	A<br>I	transposase<br>classical
**267.**	Cl107	Urine	NZ_CP098521.1/CP098521.1				
**268.**	Cl415	Blood	NZ_CP071763.1/CP071763.1		-		
**269.**	CMC-CR-MDR-Ab4	Bronchoalveolar lavage	NZ_CP016295.1/CP016295.1				
**270.**	CMC-CR-MDR-Ab66	Hospital-aquired infections	NZ_CP016300.1/CP016300.1				
**271.**	CMC-MDR-Ab59	Sputum	NZ_CP016298.1/CP016298.1				
**272.**	CUVET-MIC596	Dog urine	NZ_CP041148.1/CP041148.1				
**273.**	D1279779	Clinical	NC_020547.2/CP003967.2		-		
**274.**	D36	Wound	CP012952.1	ALJ87841.1<br>ALJ87858.1	1,995,421..1,996,665<br>2,006,581..2,007,825	C<br>C	flanked<br>flanked
**275.**	D4	Wound	NZ_CP048849.1/CP048849.1		-		
**276.**	D46	Midstream urine	NZ_CP048131.1/CP048131.1				
**277.**	DA33098	Clinical isolate	NZ_CP029569.1/CP029569.1				
**278.**	DA33382	Tracheal secretion	CP030106.1	AXB14688.1	865,926..867,170<br>857,212..857,931	C<br>pseudo	flanked
**279.**	DB002	Human isolate	NZ_CP087354.1/CP087354.1				
**280.**	DB003	Human isolate	NZ_CP087351.1/CP087351.1				
**281.**	DB006	Human isolate	NZ_CP087348.1/CP087348.1				
**282.**	DB007	Human isolate	NZ_CP087344.1/CP087344.1				
**283.**	DB008	Human isolate	NZ_CP087340.1/CP087340.1				
**284.**	DB053	Human isolate	NZ_CP087335.1/CP087335.1				
**285.**	DD520	Hospital strain	NZ_CP075321.1/CP075321.1				
**286.**	DETAB-C9	Sputum	CP104295.1				
**287.**	DETAB-E108	Bedside hospital table	NZ_CP077826.1/CP077826.1				
**288.**	DETAB-E155	Infusion stand	NZ_CP077843.1/CP077843.1				

**289.**	DETAB-E159	Locker	NZ_CP077837.1/CP077837.1				
**290.**	DETAB-E227	Human isolate	NZ_CP072526.1/CP072526.1				
**291.**	DETAB-E351	Ventilator shelf	NZ_CP077828.1/CP077828.1				
**292.**	DETAB-E51	Bed controller	NZ_CP077830.1/CP077830.1	UQY02819.1	2,279,265..2,280,521	I	classical
**293.**	DETAB-P2	Rectal swab	CP047973.1	QMS83345.1<br>QMS83424.1	3,376,853..3,377,917<br>3,459,528..3,460,991	A<br>D	transposase<br>transposase
**294.**	DETAB-P24	Nasogastric tube	NZ_CP077846.1/CP077846.1				
**295.**	DETAB-P39	Patient	NZ_CP073060.1/CP073060.1				
**296.**	DETAB-P43	Nasojejunal tube	NZ_CP077832.1/CP077832.1	UQY10098.1	1,959,551..1,960,807	I	classical
**297.**	DETAB-P65	Rectal swab	NZ_CP077835.1/CP077835.1				
**298.**	DETAB-P90	Rectal swab	NZ_CP077840.1/CP077840.1				
**299.**	DETAB-R21	Patient	NZ_CP088895.1/CP088895.1				
**300.**	DS002	Soil	CP027704.2				
**301.**	DSM30011-VUB	Plant microbiota	NZ_CP091333.1/CP091333.1	UMO44486.1<br>UMO44493.1<br>UMO44503.1	1,428,808-1,430,040<br>1,435,067-1,436,299<br>1,443,430..1,444,902	Not grouped<br>J<br>J	flanked<br>classical<br>flanked
**302.**	DT-Ab003	Stethoscope	NZ_CP050916.1/CP050916.1				
**303.**	DT-Ab007	Sputum	NZ_CP050914.1/CP050914.1				
**304.**	DT-Ab020	Sputum	NZ_CP050911.1/CP050911.1				
**305.**	DT-Ab022	Sputum	NZ_CP050907.1/CP050907.1				
**306.**	DT-Ab057	Bronchoalveolar lavage fluid	NZ_CP050904.1/CP050904.1		-		
**307.**	DT01139C	Clinical sample	NZ_CP053218.1/CP053218.1				
**308.**	DT0544C	Clinical sample	CP053215.1	QLI37522.1	3,415,257..3,416,321	A	transposase
**309.**	DU202	Sputum	NZ_CP017152.1/CP017152.1		-		
**310.**	E-011922	Paper mill kaolin	NZ_CP059542.1/CP059542.1				
**311.**	E-072658	Paper pulp mill	NZ_CP061705.1/CP061705.1				
**312.**	E47	Room 7	NZ_CP042556.1/CP042556.1				
**313.**	EAB1	Lab-evolved strain	NZ_CP112859.1/CP112859.1				
**314.**	EAB2	Lab-evolved strain	NZ_CP112860.1/CP112860.1				
**315.**	LEV1449/17Ec	Imported food and clinicalsamples	NZ_CP038262.1/CP038262.1				
**316.**	39741	Imported food and clinicalsamples	NZ_CP038258.1/CP038258.1				
**317.**	Ex003	Water (artesian well)	NZ_CP049314.1/CP049314.1				
**318.**	F-1629	Human isolate	CP099969.1				
**319.**	F46	Clinical	NZ_CP096575.1/CP096575.1				
**320.**	FDAARGOS_1036	ATCC:BAA-1605D-5	CP066016.1	QQB67876.1<br>QQB67884.1	894,409..895,584<br>900,586..901,752<br>912,170..912,889	E<br>C<br>pseudo	flanked<br>classical
**321.**	FDAARGOS_1359	Unknown	NZ_CP069851.1/CP069851.1				
**322.**	FDAARGOS_1360	Unknown	NZ_CP069840.1/CP069840.1				
**323.**	FDAARGOS_533	Sputum	CP033768.1	AYY54866.1	3,615,966..3,617,429	D	transposase
**324.**	FDAARGOS_540	Unknown	CP033754.1	AYX87484.1<br>AYX87491.1	2,586,520..2,587,638<br>2,595,642..2,596,883	D<br>C	incomplete<br>incomplete

**325.**	G20AB007	Blood	NZ_CP066237.1/CP066237.1				
**326.**	G20AB009	Blood	NZ_CP066235.1/CP066235.1				
**327.**	G20AB010	Blood	NZ_CP066232.1/CP066232.1				
**328.**	G20AB011	Blood	NZ_CP066229.1/CP066229.1				
**329.**	HKU3	Hospital	NZ_CP084733.1/CP084733.1				
**330.**	HKU4	Hospital	NZ_CP084730.1/CP084730.1				
**331.**	HKU5	Hospital	NZ_CP084727.1/CP084727.1				
**332.**	HKU6	Hospital	NZ_CP084724.1/CP084724.1				
**333.**	HKU7	Hospital	NZ_CP084721.1/CP084721.1				
**334.**	HRAB-85	Sputum	NZ_CP018143.1/CP018143.1				
**335.**	HWBA8	Sputum	NZ_CP020597.1/CP020597.1				
**336.**	IOMTU433	Respiratory tract	NZ_AP014649.1/AP014649.1				
**337.**	J9	Clinical sample	NZ_CP041587.1/CP041587.1				
**338.**	JBA13	Sputum	NZ_CP020584.1/CP020584.1				
**339.**	K09-14	Soil	NZ_CP043953.1/CP043953.1		-		
**340.**	KAB01	Bronchial	NZ_CP017642.1/CP017642.1				
**341.**	KAB02	Bronchial	NZ_CP017644.1/CP017644.1				
**342.**	KAB03	Sputum	NZ_CP017646.1/CP017646.1				
**343.**	KAB04	Sputum	NZ_CP017648.1/CP017648.1				
**344.**	KAB05	Blood	NZ_CP017650.1/CP017650.1				
**345.**	KAB06	Wound	NZ_CP017652.1/CP017652.1				
**346.**	KAB07	Sputum	NZ_CP017654.1/CP017654.1				
**347.**	KAB08	Wound	NZ_CP017656.1/CP017656.1				
**348.**	KBN10P02143	Pus	NZ_CP013924.1/CP013924.1		-		
**349.**	KBN10P04593	Blood	NZ_CP099989.1/CP099989.1				
**350.**	KBN10P05679	Human isolate	NZ_CP100305.1/CP100305.1				
**351.**	KSK1	Respiratory specimen	NZ_CP072122.1/CP072122.1				
**352.**	KSK10	Pus	NZ_CP072280.1/CP072280.1				
**353.**	KSK11	Respiratory	CP072285.1/CP072285.1				
**354.**	KSK18	Respiratory	NZ_CP072290.1/CP072290.1				
**355.**	KSK19	Respiratory	NZ_CP072295.1/CP072295.1				
**356.**	KSK2	Respiratory	NZ_CP072398.1/CP072398.1				
**357.**	KSK20	Respiratory	NZ_CP072300.1/CP072300.1				
**358.**	KSK6	Respiratory	NZ_CP072270.1/CP072270.1		-		
**359.**	KSK7	Respiratory	NZ_CP072275.1/CP072275.1				
**360.**	KSK Sensitive	Respiratory	CP072305.1	QTK75658.1	3,335,988..3,337,052	A	transposase
**361.**	LAC-4	Unknown	NZ_CP007712.1/CP007712.1				
**362.**	LAC4	Unknown	NZ_CP018677.1/CP018677.1				
**363.**	LHC22-2	Chicken feces	CP084297.1				
**364.**	LMG994	Human isolate	NZ_CP087331.1/CP087331.1	WP_140947982.1<br>WP_031986077.1	2,070,332..2,071,555<br>2,836,913..2,838,013	B<br>Not grouped	flanked<br>transposase

				WP_000585016.1	3,654,254..3,655,369	A	trasposase
**365.**	LUH 6011	Human isolate	NZ_CP031383.1/CP031383.1				
**366.**	M164-3	*Manis javanica*	NZ_CP058729.1/CP058729.1				
**367.**	M175-3	*Manis javanica*	NZ_CP059474.1/CP059474.1				
**368.**	MDR-CQ	Human isolate	NZ_CP019114.1/CP019114.1				
**369.**	MDR-TJ	Hospital sample	NC_017847.1/CP003500.1		-		
**370.**	MDR-UNC	right leg muscle, right leg fascia, central venous catheter, dialysis catheter, surface of the left leg ulcer, and left lower leg	NZ_CP031444.1/CP031444.1				
**371.**	MDR-ZJ06	Clinical	NC_017171.2/CP001937.2		-		
**372.**	MRSN 56	hip	NZ_CP080452.1/CP080452.1<br>CP090606.1				
**373.**	MRSN 58	Hip	NZ_CP090607.1/CP090607.1				
**374.**	MRSN15313	Cerebrospinal fluid	CP033869.1	AYY89232.1<br>AYY89244.1	2,210,730..2,211,902<br>2,218,293..2,219,465	F<br>F	classical<br>classical
**375.**	MRSN57	Hip	NZ_CP091172.1/CP091172.1				
**376.**	MS14413	Urine (catheter)	NZ_CP054302.1/CP054302.1				
**377.**	N13-03449	Patients from Canadian acute care hospitals	NZ_CP043417.1/CP043417.1				
**378.**	NCCP 15989	Sputum	CP099784.1				
**379.**	NCCP 15992	Human isolate	CP099786.1				
**380.**	NCCP 15995	Human isolate	CP099788.1				
**381.**	NCCP 15996	Urine	CP099790.1				
**382.**	NCCP 16006	Human isolate	CP099793.1				
**383.**	NCCP 16007	Urine	NZ_CP091465.1/CP091465.1				
**384.**	NCCP 16011	Human isolate	CP099795.1				
**385.**	NCGM 237	Respiratory, urine andblood	NZ_AP013357.1/AP013357.1		-		
**386.**	NCIMB 8209	Aerobic microbial decomposition of whole and defoliated guayule (retting)	NZ_CP028138.1/CP028138.1				
**387.**	NCTC13421	Unknown	LS483472.1	SQI54944.1<br>SQI54905.1	1,942,021..1,943,187<br>1,933,632..1,934,807	C<br>E	flanked<br>flanked
**388.**	NCTC7364	Unknown	LT605059.1	SCD14470.1<br>SCD16021.1<br>SCD16029.1	419,790..421,253<br>2,063,045..2,064,208<br>2,069,398..2,070,561	D<br>B<br>B	transposase<br>classical<br>flanked
**389.**	NIPH17_0019	Blood	NZ_AP024415.1/AP024415.1				
**390.**	Nord4-2	Human isolate	NZ_CP091596.1/CP091596.1				
**391.**	NY13623	Sputum	NZ_CP106988.1/CP106988.1	UYF87871.1	2,027,025..2,028,239	B	flanked
**392.**	NY5301	Ascites	NZ_CP094283.1/CP094283.1				
**393.**	OC043	Human isolate	NZ_CP087321.1/CP087321.1				
**394.**	OC059	Human isolate	NZ_CP087312.1/CP087312.1				

**395.**	OC061	Human isolate	NZ_CP087300.1/CP087300.1				
**396.**	OC064	Human isolate	NZ_CP087317.1/CP087317.1		2,178,662..2,179,726	A	flanked
**397.**	OC068	Human isolate	NZ_CP087309.1/CP087309.1				
**398.**	OC070	Human isolate	NZ_CP087298.1/CP087298.1				
**399.**	OC073	Human isolate	NZ_CP087325.1/CP087325.1		3,355,470..3,356,957	D	trasposase
**400.**	OC074	Human isolate	NZ_CP087328.1/CP087328.1		1,906,934..1,908,100	C	flanked
**401.**	OC081	Human isolate	NZ_CP087304.1/CP087304.1		1,908,368..1,909,612<br>1,917,220..1,917,371	C<br>pseudo	flanked
**402.**	OCU_Ac16a	Tracheal aspirate from a hospitalized patient	AP023077.1		1,863,263..1,864,738	D	flanked
**403.**	OCU Ac18	Venous blood	NZ_AP024802.1/AP024802.1				
**404.**	ORAB01	Bodily fluid	NZ_CP015483.1/CP015483.1				
**405.**	P7774	Pus	NZ_CP040260.1/CP040260.1				
**406.**	PB364	Clinicalisolate	NZ_CP040425.1/CP040425.1				
**407.**	PG20180064	Human isolate	NZ_CP014538.1/CP014538.1				
**408.**	PM1912235	Pus	NZ_CP050410.1/CP050410.1				
**409.**	PM192696	Sputum	NZ_CP050412.1/CP050412.1				
**410.**	PM193665	Pus	NZ_CP050415.1/CP050415.1				
**411.**	PM194188	Bronchoalveolar lavage	NZ_CP050425.1/CP050425.1				
**412.**	PM194229	Bronchoalveolar lavage	NZ_CP050432.1/CP050432.1				
**413.**	R2090	Tissue	NZ_LN868200.1/LN868200.1				
**414.**	R2091	Tissue	NZ_LN997846.1/LN997846.1	CUW33887.1<br>CUW35246.1	491,918..492,982<br>1,965,798..1,967,051	A<br>I	transposase<br>classical
**415.**	RBH2	Human isolate	CP110462.1				
**416.**	RCH52	Unknown	NZ_CP085788.1/CP085788.1				
**417.**	Res13-Abat-PEA21-P4-01-A	Pigswab	NZ_CP062919.1/CP062919.1				
**418.**	S1, AB1A2	Bronchoalveolar lavage	NZ_CP026943.1/CP026943.1		-		
**419.**	SAA14	Blood	NZ_CP020579.1/CP020579.1				
**420.**	SD	Clinical isolate	CP064292.1		-		
**421.**	SHOU-Ab01	*Andrias davidianus*	NZ_CP087594.1/CP087594.1				
**422.**	SMC_Paed_Ab_BL01	Blood, central line	NZ_CP025266.1/CP025266.1				
**423.**	SP304	Sputum	NZ_CP040080.1/CP040080.1				
**424.**	SSA12	Blood	NZ_CP020578.1/CP020578.1				
**425.**	SSA6	Endotracheal aspirate	NZ_CP020591.1/CP020591.1				
**426.**	SSMA17	Bronchial washing fluid	NZ_CP020581.1/CP020581.1				
**427.**	TCDC-AB0715	Unknown	CP002522.2		-		
**428.**	TG22182	Tracheal aspirate	NZ_CP039993.1/CP039993.1				
**429.**	TG22627	Tracheal aspirate	NZ_CP039520.1/CP039520.1				
**430.**	TG22653	Bronchoalveolar lavage	NZ_CP039518.1/CP039518.1				
**431.**	TG29392	Blood	NZ_CP039930.1/CP039930.1				
**432.**	TG31302	Tracheal aspirate	NZ_CP039343.1/CP039343.1				
**433.**	TG31986	Tracheal aspirate	NZ_CP039341.1/CP039341.1				

**434.**	TP1	Human clinical sample	NZ_CP056784.2/CP056784.2				
**435.**	TP2	Human clinical sample	NZ_CP060011.1/CP060011.1		-		
**436.**	TP3	Human clinical sample	NZ_CP060013.1/CP060013.1				
**437.**	TYTH-1	Bacteremia	NC_018706.1/CP003856.1		-		
**438.**	UC20804	Peritoneal abcess	NZ_CP076807.1/CP076807.1				
**439.**	UC21460	Tissue	NZ_CP076814.1/CP076814.1	UTA05877.1	1,824,561..1,825,775	B	flanked
**440.**	UC22850	Catheter tip	NZ_CP076821.1/CP076821.1	UTA10383.1<br>UTA10372.1	2,501,931..2,503,103<br>2,494,511..2,495,755	F<br>C	classical<br>flanked
**441.**	UC23022	Blood culture	NZ_CP076812.1/CP076812.1	UTA01520.1<br>UTA01528.1<br>UTA01540.1	1111264..1112478<br>1117290..1118462<br>1124853..1126025	B<br>F<br>F	flanked<br>classical<br>classical
**442.**	UC24137	Sacrum ulcer	NZ_CP076817.1/CP076817.1				
**443.**	UC24371	Endotracheal aspirate	NZ_CP076804.1/CP076804.1				
**444.**	UC25604	Peritoneal liquid	NZ_CP076801.1/CP076801.1				
**445.**	USA15	Sputum	CP020595.1	ARG31808.1	2,251,549..2,252,715	C	flanked
**446.**	USA2	Urine	NZ_CP020592.1/CP020592.1				
**447.**	VB11737	Blood	NZ_CP050400.1/CP050400.1				
**448.**	VB1190	Blood	NZ_CP040047.1/CP040047.1				
**449.**	VB16141	Blood	CP040050.1	QCP31132.1	1,853,393..1,854,553	G	flanked (pseudo)
**450.**	VB2107	Sputum	NZ_CP051474.1/CP051474.1				
**451.**	VB2139	Sputum	NZ_CP050526.1/CP050526.1				
**452.**	VB2181	Sputum	NZ_CP050401.1/CP050401.1				
**453.**	VB2200	Sputum	NZ_CP050421.1/CP050421.1				
**454.**	VB23193	Blood	CP035672.1		-		
**455.**	VB2486	Sputum	CP050403.1	QJH02942.1<br>QJH02928.1	2,036,912..2,038,156<br>2,027,603..2,028,778	C<br>E	flanked<br>flanked
**456.**	VB280821	Blood	NZ_CP098795.1/CP098795.1	USI43228.1<br>USI43236.1<br>USI41163.1	644,586-645,464<br>652,274..653,338<br>2,317,985..2,319,049	I<br>A<br>A	transposase<br>flanked<br>transposase
**457.**	VB31459	Blood	CP035930.1		-		
**458.**	VB33071	Blood	CP040084.1/CP040084.1				
**459.**	VB35179	Blood	CP040053.1		-		
**460.**	VB35435	Blood	CP040056.1		-		
**461.**	VB35575	Blood	NZ_CP040087.1/CP040087.1				
**462.**	VB473	Sputum	NZ_CP050388.1/CP050388.1				
**463.**	VB7036	Blood	NZ_CP050523.1/CP050523.1				
**464.**	VB723	Blood	NZ_CP050390.1/CP050390.1				
**465.**	VB82	Blood	NZ_CP050385.1/CP050385.1		-		
**466.**	VB958	Blood	CP040040.1		-		
**467.**	WCHAB005078	Human isolate	CP027246.2	AVN14434.1	1,910,050..1,911,294	C	flanked
**468.**	WCHAB005133	Human isolate	NZ_CP026750.2/CP026750.2				

**469.**	WKA02	Sputum	NZ_CP020598.1/CP020598.1				
**470.**	WP4-W18-ESBL-11	Wastewater treatment plant effluent	AP022077.1	BBR72034.1<br>BBR73561.1	390,603..392,090<br>2,006,965..2,008,209	D<br>C	transposase flanked
**471.**	XDR-BJ83	Medical male patient	NZ_CP018421.1/CP018421.1				
**472.**	XH1056	Sputum	NZ_CP045645.1/CP045645.1	USR84134.1	2,124,779..2,125,849	F	Classical (incomplete)
**473.**	XH1344	Yunsongyu'sLab	CP061541.1	QNV46553.1	1,982,136..1,983,365	B	flanked (pseudo)
**474.**	XH1935	Patient	NZ_CP088894.1/CP088894.1				
**475.**	XH386	Lower respiratory tract	NZ_CP010779.1/CP010779.1<br>NZ_CP021326.1/CP021326.1		-		
**476.**	XH731	Sputum	NZ_CP019217.1/CP019217.1<br>NZ_CP021321.1/CP021321.1				
**477.**	XH856	Drainage fluid	NZ_CP014541.1/CP014541.1				
**478.**	XH857	Sputum	NZ_CP014540.1/CP014540.1				
**479.**	XH858	Sputum	NZ_CP014528.1/CP014528.1	AMN01582.1	2,163,381..2,164,451	F	classical (incomplete)
**480.**	XH859	Wound	NZ_CP014539.1/CP014539.1				
**481.**	XH860	Human clinical isolate	NZ_CP014538.1/CP014538.1				
**482.**	XH906	Blood	NZ_CP023140.1/CP023140.1				
**483.**	XL380	Cucumber rhizosphere	NZ_CP046536.1/CP046536.1				
**484.**	XYAB2018	Blood	NZ_CP060285.1/CP060285.1				
**485.**	YC103	Stool	NZ_CP054560.1/CP054560.1	UJX51745.1	1,932,409..1,932,810	G (incomplete)	classical
**486.**	YU-R612	Sputum	NZ_CP014215.1/CP014215.1				
**487.**	YZM-0314	Bacteremia	NZ_CP104908.1/CP104908.1				
**488.**	YZM-0406	Bacteremia	NZ_CP104912.1/CP104912.1				
**489.**	Z198	Cerebrospinal fluid	NZ_CP109836.1/CP109836.1				
**490.**	ZW85-1	Feces	NC_023028.1/CP006768.1				
**491.**	04117201	Clinicalisolate	CP079943.1<br>CP079944.1				
**492.**	2022CK-00843	Human isolate	CP117759.1				
**493.**	RAB9	Clinical isolate	NZ_CP121557.1/CP121557.1				
**494.**	AB233-VUB	Clinical isolate	NZ_CP091336.1/CP091336.1				
**495.**	2022CK-00063	Sputum	CP115632.1				
**496.**	RAB94	Clinical isolate	NZ_CP121563.1/CP121563.1				
**497.**	RAB55	Clinical isolate	NZ_CP121577.1/CP121577.1				
**498.**	A9844	Clinical isolate	CP102580.1				
**499.**	96	Bronchial secretion	NZ_CP121345.1/CP121345.1				
**500.**	2022CK-00783	Urine	CP117728.1				
**501.**	2022CK-00251	Sputum	CP115641.1				
**502.**	2022CK-00211	Sputum	CP115637.1				

**503.**	2022CK-00371	Skin	CP115645.1				
**504.**	AB1343	Sputum	NZ_CP090182.1/CP090182.1				
**505.**	EGA65	Peripheral blood	NZ_CP125223.1/CP125223.1				
**506.**	EGA10	Hip wound	NZ_CP125225.1/CP125225.1				
**507.**	RAB73	Clinical isolate	NZ_CP121567.1/CP121567.1				
**508.**	2022CK-00241	Sputum	CP115639.1				
**509.**	OCU-Ac19	Clinical isolate	AP025531.1				
**510.**	2022CK-00185	Human isolate	CP115626.1				
**511.**	HAB11	Clinical isolate	NZ_CP121632.1/CP121632.1				
**512.**	2022CK-00480	Sputum	CP115621.1				
**513.**	2022CK-00340	Wound/abscess	CP115623.1				
**514.**	RAB14	Clinical isolate	NZ_CP121583.1/CP121583.1				
**515.**	RAB73	Clinical isolate	NZ_CP121567.1/CP121567.1				
**516.**	WU 164	Urine	NZ_CP033876.1/CP033876.1				
**517.**	X4-65	Human isolate	NZ_CP064194.1/CP064194.1				
**518.**	X4-300	Human isolate	CP064202.1				
**519.**	X4-136	Human isolate	CP074698.1				
**520.**	X4-107	Human isolate	CP074695.1				
**521.**	X4-201	Blood	CP076736.1				
**522.**	X4-584	Urine	CP076739.1				
**523.**	X4-705	Pleural effusion	CP076742.1				
**524.**	MAB17	Clinicalisolates	NZ_CP121595.1/CP121595.1				
**525.**	Canada BC-5	Nosocomial spread of war-related MDR-ABC in a Canadian civilian hospital with origin from soldier evacuated via Landstuhl Regional Medical Center	NZ_CP116680.1/CP116680.1				
**526.**	WB4	Feedlot Water bowl	CP123854.1				
**527.**	2022CK-00784	Wound/abscess	CP117764.1				
**528.**	Ab4294	Human isolate	CP123993.1				
**529.**	WP8-W18-ESBL-11	Wastewater treatment plant effluent	NZ_AP022238.1/AP022238.1				
**530.**	LRT	Wastewater	NZ_CP121375.1/CP121375.1				
**531.**	LRB	Wastewater	NZ_CP121370.1/CP121370.1				
**532.**	Ab_8_4	Wastewater	NZ_CP121365.1/CP121365.1				
**533.**	RAB97	Clinical isolate	NZ_CP121560.1/CP121560.1				
**534.**	RAB53	Clinical isolate	NZ_CP121579.1/CP121579.1				
**535.**	RAB11	Clinical isolate	NZ_CP121586.1/CP121586.1				
**536.**	JAB270	Clinical isolate	NZ_CP121629.1/CP121629.1				
**537.**	JAB144	Clinical isolate	NZ_CP121625.1/CP121625.1				
**538.**	JAB117	Clinical isolate	NZ_CP121612.1/CP121612.1				
**539.**	JAB186	Clinical isolate	NZ_CP121609.1/CP121609.1				

**540.**	JAB77	Clinical isolate	NZ_CP121598.1/CP121598.1				
**541.**	MAB9	Clinical isolate	NZ_CP121588.1/CP121588.1				

[1]flanked – prophage genome flanked on both sides by replication/regulation module; [2]classical – prophage with morphogenesis, structural and replicative/regulatory; [3]transposase – a transposase in addition to classical genes
